# Genotype and Lifetime Burden of Disease in Hypertrophic Cardiomyopathy: Insights From the Sarcomeric Human Cardiomyopathy Registry (SHaRe)

**DOI:** 10.1161/CIRCULATIONAHA.117.033200

**Published:** 2018-10-01

**Authors:** Carolyn Y. Ho, Sharlene M. Day, Euan A. Ashley, Michelle Michels, Alexandre C. Pereira, Daniel Jacoby, Allison L. Cirino, Jonathan C. Fox, Neal K. Lakdawala, James S. Ware, Colleen A. Caleshu, Adam S. Helms, Steven D. Colan, Francesca Girolami, Franco Cecchi, Christine E. Seidman, Gautam Sajeev, James Signorovitch, Eric M. Green, Iacopo Olivotto

**Affiliations:** 1 Cardiovascular Division, Brigham and Women’s Hospital, Boston, MA (C.Y.H., A.L.C., N.K.L.).; 2 Department of Internal Medicine, University of Michigan, Ann Arbor (S.M.D., A.S.H., C.E.S.).; 3 Stanford Center for Inherited Heart Disease, CA (E.A.A., C.A.C.).; 4 Department of Cardiology, Thoraxcenter, Erasmus Medical Center Rotterdam, the Netherlands (M.M.).; 5 Heart Institute (InCor), University of Sao Paulo Medical School, Brazil (A.C.P.).; 6 Yale University, New Haven, CT (D.J.).; 7 MyoKardia, Inc, South San Francisco, CA (J.D.F., E.M.G.).; 8 National Heart and Lung Institute and National Institute for Health Research Royal Brompton Cardiovascular Biomedical Research Unit, Imperial College London, United Kingdom (J.S.W.).; 9 Department of Cardiology, Boston Children’s Hospital, MA (S.D.C.).; 10 Cardiomyopathy Unit and Genetic Unit, Careggi University Hospital, Florence, Italy (F.G., F.C., I.O.).; 11 Howard Hughes Medical Institute, Chevy Chase, MD (C.E.S.).; 12 Analysis Group Inc, Boston, MA (G.S., J.S.).

**Keywords:** cardiomyopathy, hypertrophic, genetics, natural history, registries, risk

## Abstract

**Background:**

A better understanding of the factors that contribute to heterogeneous outcomes and lifetime disease burden in hypertrophic cardiomyopathy (HCM) is critically needed to improve patient management and outcomes. The SHaRe registry (Sarcomeric Human Cardiomyopathy Registry) was established to provide the scale of data required to address these issues, aggregating longitudinal data sets curated by 8 international HCM specialty centers.

**Methods:**

Data on 4591 patients with HCM (2763 genotyped) followed up for a mean of 5.4±6.9 years (24 791 patient-years; median, 2.9 years; interquartile range, 0.3–7.9 years) were analyzed for cardiac arrest, cardiac transplantation, appropriate implantable cardioverter-defibrillator therapy, all-cause death, atrial fibrillation, stroke, New York Heart Association functional class III/IV symptoms (all making up the overall composite end point), and left ventricular ejection fraction <35%. Outcomes were analyzed individually and as composite end points.

**Results:**

Median age at diagnosis was 45.8 (interquartile range, 30.9–58.1) years, and 37% of patients were female. Age at diagnosis and sarcomere mutation status were predictive of outcomes. Patients <40 years old at diagnosis had a 77% (95% CI, 72–80) cumulative incidence of the overall composite outcome by 60 years of age compared with 32% (95% CI, 29–36) by 70 years of age for patients diagnosed at >60 years old. Young patients with HCM (age, 20–29 years) had 4-fold higher mortality than the general US population at a similar age. Patients with pathogenic/likely pathogenic sarcomere mutations had a 2-fold greater risk for adverse outcomes compared with patients without mutations; sarcomere variants of uncertain significance were associated with intermediate risk. Heart failure and atrial fibrillation were the most prevalent adverse events, although typically not emerging for several years after diagnosis. Ventricular arrhythmias occurred in 32% (95% CI, 23–40) of patients <40 years of age at diagnosis but in 1% (95% CI, 1–2) of those >60 years old at diagnosis.

**Conclusions:**

The cumulative burden of HCM is substantial and dominated by heart failure and atrial fibrillation occurring many years after diagnosis. Young age at diagnosis and the presence of a sarcomere mutation are powerful predictors of adverse outcomes. These findings highlight the need for close surveillance throughout life and the need to develop disease-modifying therapies.

## Abstract 2

Supplemental Digital Content is available in the text.

**Clinical Perspective**

**What Is New?**

- This study analyzed longitudinal clinical information on 4591 patients with hypertrophic cardiomyopathy (HCM) who receive care at 8 high-volume multinational HCM specialty centers.
- In an examination of a data set spanning >24 000 patient-years, the mortality of patients with HCM was shown to be ≈3-fold higher than that of the US general population at similar ages.
- The lifetime cumulative morbidity of HCM was considerable, particularly for patients diagnosed before 40 years of age and patients with sarcomere mutations.
- Atrial fibrillation and heart failure were the dominant components of disease burden.
- This work highlights the power of international collaboration to study complex and heterogeneous disease.

**What Are the Clinical Implications?**

- Younger age at diagnosis and the presence of a sarcomere mutation were strong predictors of adverse events.
- Considering these factors will likely improve prognostication and risk stratification for individual patients with HCM.
- Although the incidence of ventricular arrhythmias declined with age, the risk for heart failure and atrial fibrillation increased, becoming most prevalent by mid- to late adulthood, even in patients diagnosed before 40 years of age.
- These observations underscore the need for lifelong surveillance and for developing effective strategies to prevent the progressive and adverse remodeling that leads to these complications.

**Editorial, see p 1399**

Hypertrophic cardiomyopathy (HCM) is a primary myocardial disorder defined by left ventricular (LV) hypertrophy that occurs in the absence of identifiable processes that could account for such remodeling. HCM has been the focus of intense clinical and basic science investigation for decades. These efforts have provided remarkable insights into both the molecular basis and clinical course of disease, defining sarcomere mutations as the most common genetic basis of HCM^1^ and improving understanding from the early perception of a highly mortal condition.^2–4^ Although disease course is variable, many patients experience adverse clinical outcomes, including heart failure (HF), arrhythmias, and sudden cardiac death. Marked clinical heterogeneity and limited longitudinal data, particularly on large genotyped cohorts, have been major barriers to gaining an accurate and comprehensive description of the natural history of HCM. Determining the relationship among genotype, phenotype, and outcomes over a lifetime is critical to improve risk stratification and to guide patient management. Such insights are also needed to support the development of targeted therapies intended to modify disease progression and to prevent adverse sequelae.

The SHaRe registry (Sarcomeric Human Cardiomyopathy Registry) is an international database of patients with primary cardiomyopathies. Detailed data sets from expert centers were centralized and harmonized to create a large, comprehensive, collaborative registry of patients with HCM. The scale of these data, spanning >24 000 patient-years, provides the opportunity to quantify cumulative disease burden in HCM, to define present-day risk estimates, and to determine how sarcomere mutations influence disease.

## Methods

### Participating Sites and Creation of the Centralized Database

SHaRe was established by 8 experienced, high-volume HCM centers (Figure IA in the online-only Data Supplement) that maintain longitudinal databases capturing clinical, genetic, and outcomes data on patients with HCM and families under care. Definitions were harmonized for key demographic, historical, clinical, and genetic parameters. Particular attention was paid to gain complete ascertainment of historical events that occurred before SHaRe entry. This was accomplished through systematic and thorough review of medical history and medical records, both at the initial visit and at subsequent visits. Site data (without protected health information) were mapped to 762 discrete corresponding fields in the secure centralized database to yield standardized SHaRe definitions and values (Boston Advanced Analytics, Boston, MA). Prospective data were captured via quarterly uploads from site databases. Institutional review board and ethics approval was obtained in accordance with policies applicable to each SHaRe site.

The data will not be made available to other researchers for purposes of reproducing the results or replicating the procedure because of constraints related to human subjects research. Analytical methods will be made available on request.

### Study Population, Genetic Testing, and Variant Classification

Inclusion criteria included a site-designated diagnosis of HCM, defined as unexplained LV hypertrophy with maximal LV wall thickness >13 mm (or equivalent *z* score for pediatric patients), integrating extracardiac features, familial context or sporadic occurrence, and genotype to allow an informed diagnosis by experienced clinicians. Inclusion also required ≥1 clinic visit at a SHaRe site since 1960 and ≥1 echocardiographic assessment of LV wall thickness (Figure IB in the online-only Data Supplement). All analyzed metrics of cardiac dimensions and function were based on echocardiographic measurements. Genetic testing was performed at all sites using different platforms available over time. Variants were classified as pathogenic, likely pathogenic, unknown significance, or likely benign/benign by each site using contemporary criteria,^5^ focusing on the 8 sarcomere genes definitively associated with HCM (myosin binding protein C [*MYBPC3*],myosin heavy chain [*MYH7*],cardiac troponin T [*TNNT2*],cardiac troponin I [*TNNI3*], α-tropomyosin [*TPM1*], myosin essential and regulatory light chains [*MLY2, MYL3*], and actin [*ACTC*]). Sarcomere variants in the SHaRe database underwent additional systematic review by a subgroup of investigators (A.L.C., S.M.D., J.S.W., C.Y.H.; Methods in the online-only Data Supplement) to adjudicate and standardize classification. Genotyped patients were then designated as SARC+ (at least 1 pathogenic or likely pathogenic variant in any of the above sarcomere genes), which includes the subsets SARC1+ (only 1 pathogenic or likely pathogenic mutation) and SARC2+ (>1 pathogenic or likely pathogenic sarcomere mutation); SARC VUS (sarcomere variant of unknown significance present); and SARC− (no potentially pathogenic variants identified in a sarcomere gene). Patients were excluded if they had potentially pathogenic variants in genes encoding nonsarcomere proteins such as α-galactosidase (*GLA*) or lysosome-associated membrane protein (*LAMP2*) indicating the presence of metabolic or storage disease or other diagnoses. Table I in the online-only Data Supplement includes all gene variants identified in SHaRe, their classification, and their frequency in SHaRe.

### Outcome Definitions

Composite outcomes were defined to organize events into clinically relevant and related groups and to maximize statistical power. Outcomes were documented by the primary cardiologist at each site during clinical encounters and captured directly into the database:

- Ventricular arrhythmic composite: first occurrence of sudden cardiac death, resuscitated cardiac arrest, or appropriate implantable cardioverter-defibrillator therapy
- HF composite: first occurrence of cardiac transplantation, LV assist device implantation, LV ejection fraction <35%, or New York Heart Association class III/IV symptoms
- Overall composite: first occurrence of any component of the ventricular arrhythmic or heart failure composite end point (without inclusion of LV ejection fraction), all-cause mortality, atrial fibrillation (AF), stroke, or death

### Statistical Analysis

Retrospective data were analyzed from the ongoing prospective registry study. Natural history was described in terms of age at first occurrence of each composite outcome and individual components with Kaplan-Meier analyses. Patients who did not have the outcome of interest were censored at the time of their last recorded follow-up in SHaRe. Patients missing data on the occurrence or timing of events were not included in analyses of those outcomes. However, to maximize sample sizes across analyses, such patients were included in analyses of other outcomes for which data were available.

Analyses were first performed in the full HCM cohort, made up of all eligible SHaRe patients. To characterize lifetime morbidity, cumulative incidence from birth of AF and the 3 composite outcomes was derived from the Kaplan-Meier analyses of these outcomes. These analyses were stratified by age at diagnosis (<40, 40–60, >60 years). In addition, the occurrence of incident events after HCM diagnosis was analyzed, and annualized postdiagnosis incidence rates of the overall composite were calculated for each age at diagnosis stratum. To contextualize mortality rates observed in patients with HCM, age-specific mortality rates from SHaRe sites in the United States derived from the Kaplan-Meier analyses were compared with mortality rates in the US general population from 1999 to 2014 obtained from the Centers for Disease Control and Prevention Wonder database (http://wonder.cdc.gov/).

Analyses were then performed in the genotyped HCM cohort, made up of patients who underwent genetic testing to determine sarcomere mutation status. Ages at first event were compared for patients stratified by sarcomere genotype using hazard ratios based on Cox proportional hazards models with family-specific frailty effects to account for correlation resulting from relatedness. Selected outcomes were also analyzed in multivariable models. Mortality was compared between patients with nonfamilial HCM (sarcomere-negative patients without a family history of HCM) and familial HCM (SARC+, SARC VUS, or family history of HCM). Age-specific mortality in the subset of patients with nonfamilial HCM was also compared with the US general population mortality as done for the full HCM cohort. Additional details are provided in Methods in the online-only Data Supplement.

## Results

### Clinical Characteristics

Among patients with HCM receiving care at a SHaRe site between 1960 and December 2016 (n=5669), 4591 met inclusion criteria and make up the full HCM cohort (Figure IB in the online-only Data Supplement). Baseline characteristics and descriptive information on outcomes are summarized in Table 1. Most individuals were probands (first family member presenting for care at the site); affected relatives made up 12% of the cohort (552 of 4591). Median (interquartile range [IQR]) time of follow-up from the time of first encounter at a SHaRe site was 2.9 (IQR, 0.3–7.9) years, representing a total of 24 791 patient-years. Median age at diagnosis was 45.8 (IQR, 30.9–58.1) years, and 37% (1704 of 4591) were female. Forty-one percent of patients (n=1834 of 4591) reached the overall composite outcome. Events were most frequently AF (20% of patients) and HF (22% of patients), whereas 6% of patients (n=270) met the ventricular arrhythmia composite.

**Table 1.**
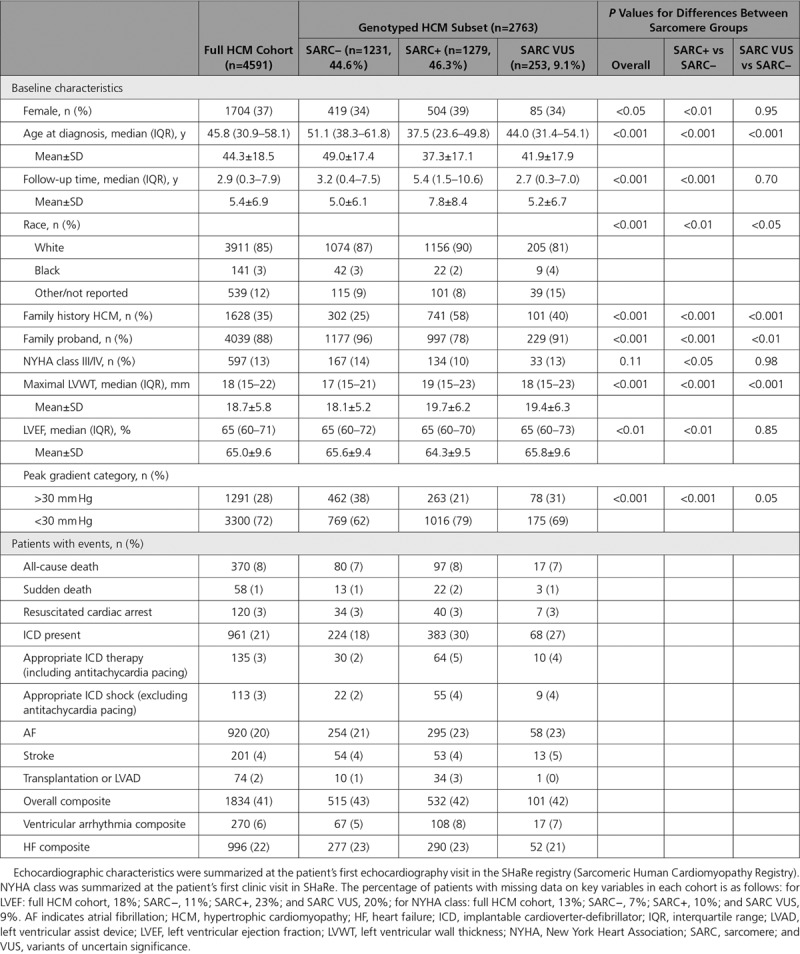
Baseline Characteristics and Events

A total of 2763 patients (60%) had genetic testing. Figure II in the online-only Data Supplement shows summary results of genetic testing in the 2405 probands tested. A pathogenic/likely pathogenic sarcomere variant was identified in 46% of all patients tested (SARC+, n=1279), including 34 subjects who carried ≥2 pathogenic/likely pathogenic variants (SARC2+); 9% had a variant of unknown significance (SARC VUS, n=253). Forty-five percent had negative genetic testing results (SARC−, n=1231). The median age at diagnosis was 13.6 years younger in SARC+ compared with SARC− patients (37.5 [IQR, 23.6–49.8] years versus 51.1 [IQR, 38.3–61.8] years; *P*<0.001).

### Natural History of HCM: Mortality and Cumulative Burden of Disease

**Figure 1.**
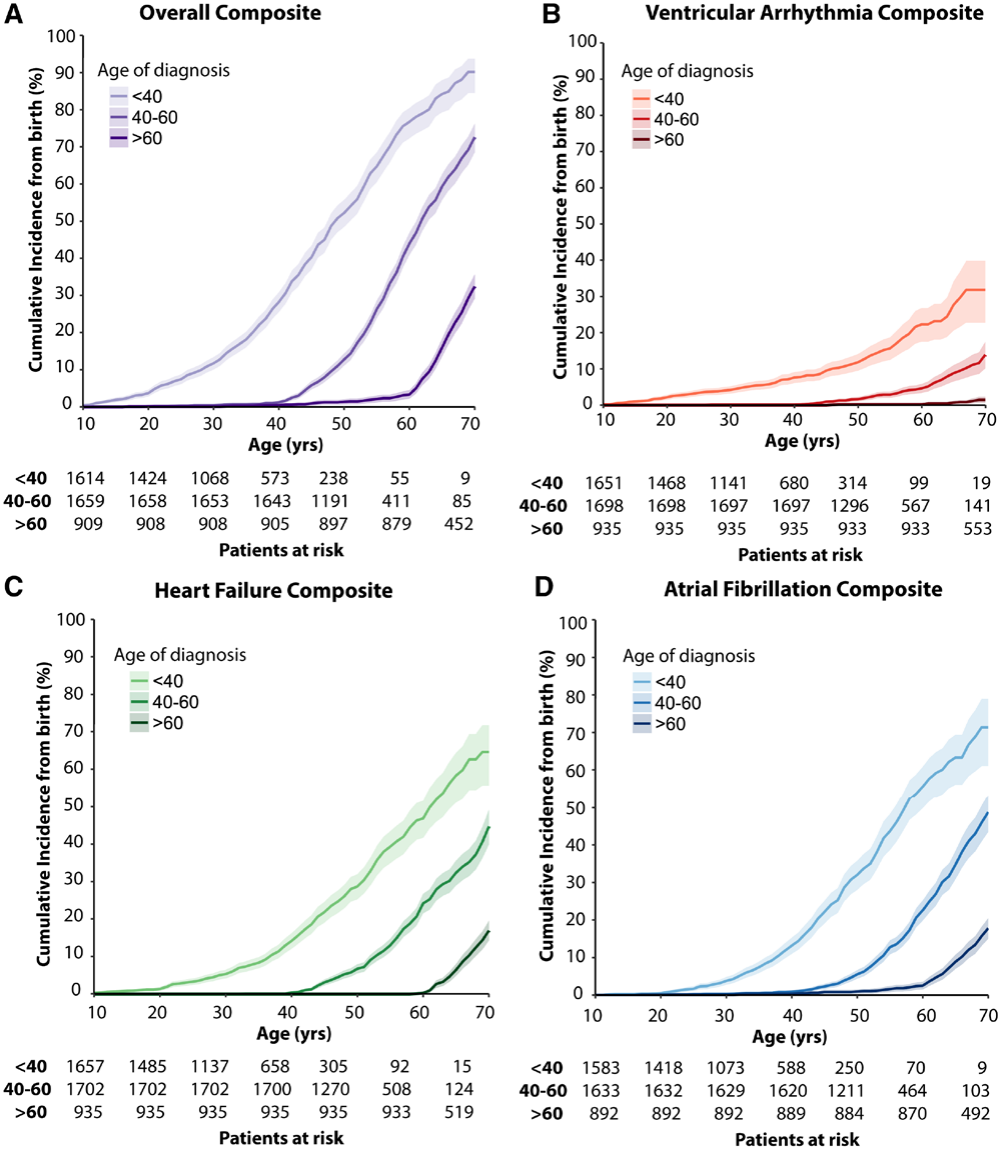
**Age at diagnosis is associated with the lifetime cumulative burden of events.** These curves depict the cumulative incidence of events from birth for outcomes of interest, stratified by age at diagnosis <40, 40 to 60, and >60 years. Earlier age at diagnosis is associated with a higher burden of adverse events. Shaded areas indicate 95% CIs. **A**, Overall composite outcome. **B**, Ventricular arrhythmia composite. **C**, Heart failure composite. **D**, Atrial fibrillation.

During follow-up, 370 patients (8%) died, including 58 sudden deaths (16% of deaths; 1% of the cohort). In addition, 120 patients had resuscitated cardiac arrest (3% of the cohort). Lifetime morbidity from HCM was analyzed by examining the cumulative incidence of events from birth to age 70 years in each age at diagnosis stratum separately (Figure 1A–1D). Younger age at diagnosis was associated with an increased cumulative incidence of events throughout life, although events largely occurred in later decades. Patients <40 years old at diagnosis had a 77% (95% CI, 72–80) cumulative incidence of the overall composite outcome by age 60 years (Figure 1A). In contrast, patients >60 years of age at diagnosis had a 32% (95% CI, 29–36) cumulative incidence of the overall composite outcome by 70 years of age. For all age at diagnosis strata, outcomes were dominated by HF (Figure 1C) and AF (Figure 1D), occurring most frequently between 50 and 70 years of age. More than 80% of patients who developed New York Heart Association class III/IV symptoms had LV ejection fraction >55% (data not shown). Lifetime cumulative incidence of malignant ventricular arrhythmias was 32% (95% CI, 23–40) in patients diagnosed at <40 years of age but was rarely encountered (1%; 95% CI, 1–2) in the oldest age at diagnosis stratum (Figure 1B). Results were comparable when analyses were repeated including only probands (data not shown), suggesting limited bias associated with ascertaining family members.

Analyses characterizing incident events occurring after the time of diagnosis demonstrated that patients diagnosed at older ages had a higher annualized risk of incident occurrence of the overall composite outcome (Table II in the online-only Data Supplement). This increased postdiagnosis risk is consistent with the steeper slope of the cumulative incidence plot for the older diagnosed patients compared with the younger diagnosed patients (Figure 1A).

**Figure 2.**
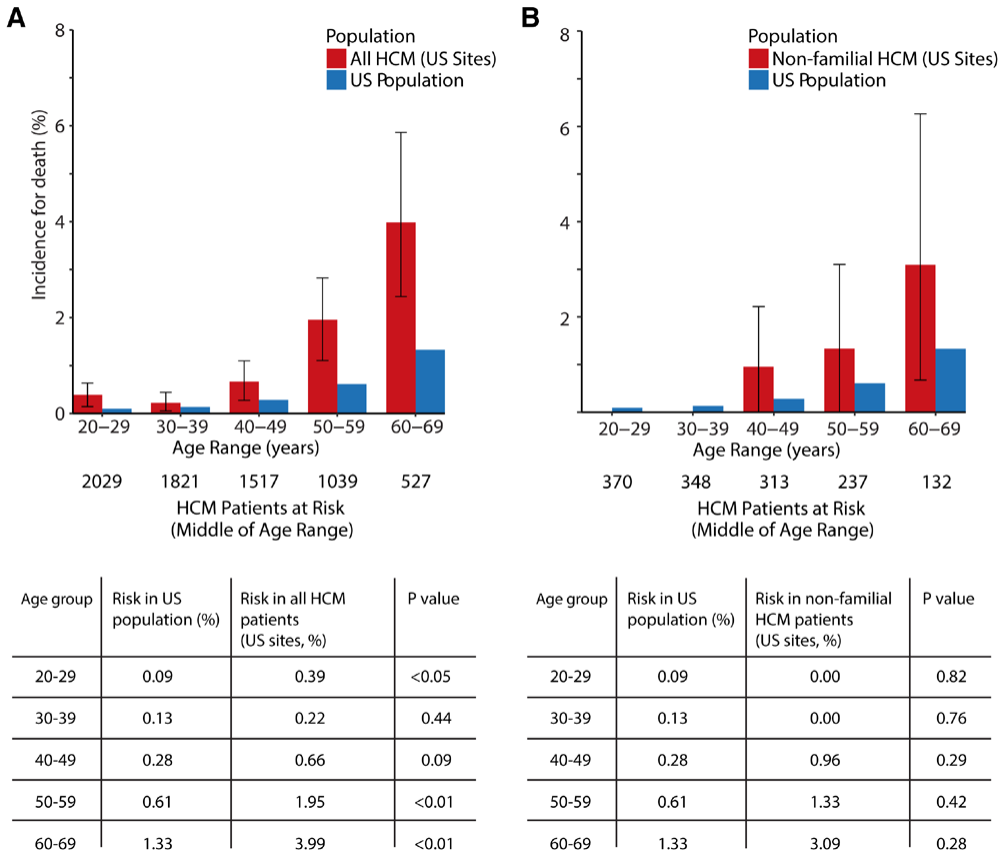
**Age-specific mortality in hypertrophic cardiomyopathy (HCM) compared with the general US population from 1999 to 2014.** Data from US SHaRe registry (Sarcomeric Human Cardiomyopathy Registry) sites were compared with the Centers for Disease Control and Prevention Wonder database (http://wonder.cdc.gov/) to estimate US general population mortality rates from 1999 to 2014. **A**, Compared with the general US population, patients with HCM have significantly increased mortality in the youngest and older age groups. **B**, When the comparison was restricted to the nonfamilial HCM cohort (no sarcomere mutation and no family history of HCM), overall mortality appears similar to that of the general US population. Error bars represent 95% CIs.

Compared with the US general population, mortality was >4-fold higher for young patients with HCM at US sites (age, 20–29 years; 0.39% versus 0.09%; *P*<0.05) and ≥3-fold higher in patients with HCM who were 50 to 69 years of age (*P*<0.01; Figure 2A). These findings of excess mortality remained comparable when patients with HCM cared for at non-US sites were included in analyses (data not shown).

### Sarcomere Mutations Are Associated With Earlier Onset and Higher Incidence of Events

**Figure 3.**
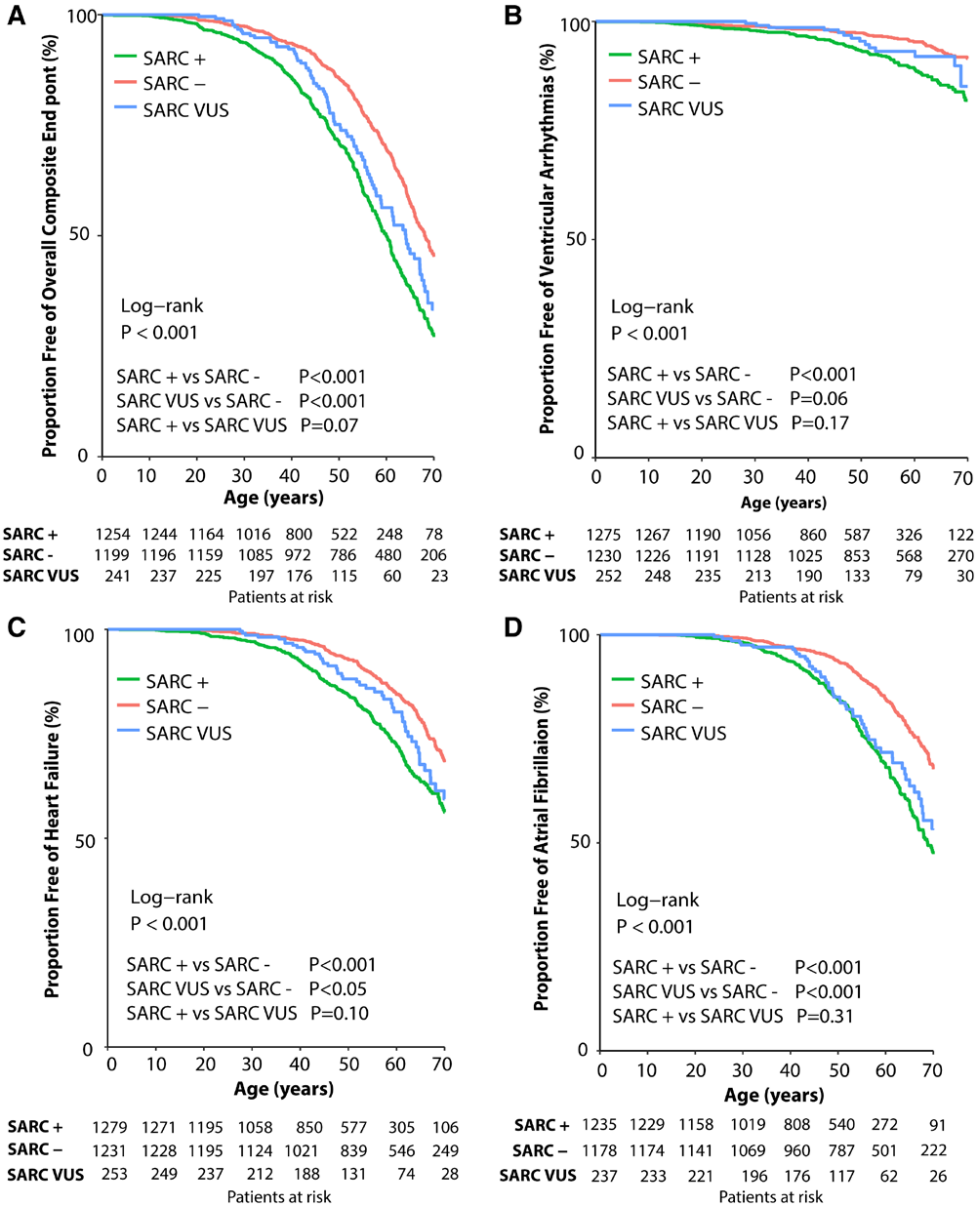
**Association between sarcomere mutations and clinical outcomes.** Kaplan-Meier survival analyses were performed in the genotyped hypertrophic cardiomyopathy (HCM) subset. Compared with patients with HCM without sarcomere mutations (SARC−), sarcomere mutation carriers (SARC+) have earlier and a higher incidence of adverse outcomes, particularly those with pathogenic and likely pathogenic variants. **A**, The risk of developing the overall composite outcome by age 50 years is 29.1% for SARC+ patients vs 24.9% for carriers of a variant of unknown significance (SARC VUS) and 14.2% for SARC− patients. **B**, Ventricular arrhythmia composite outcome. **C**, Heart failure composite outcome. **D**, Atrial fibrillation.

**Figure 4.**
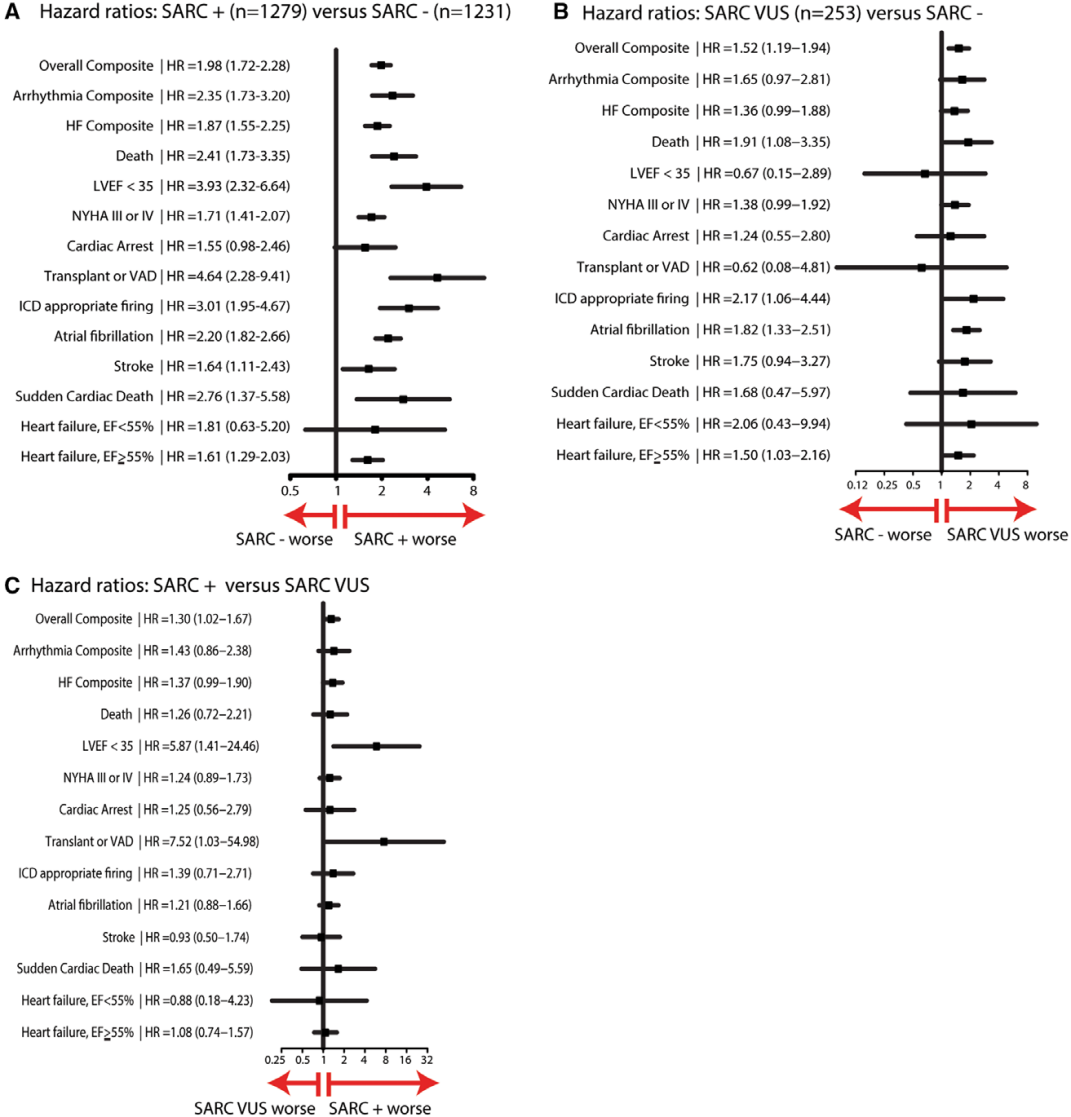
**Forest plots showing hazard ratios (HRs) for the composite end points and their individual components, comparing sarcomere mutation carriers (SARC+), patients with hypertrophic cardiomyopathy (HCM) without sarcomere mutations (SARC−), and carriers of a variant of unknown significance (SARC VUS) cohorts. A**, SARC+ vs SARC−: Sarcomere mutation carriers have a higher risk of all individual components of the composite end points compared with patients with HCM without sarcomere mutations. **B**, SARC VUS vs SARC−: SARC VUS patients have a higher risk of the overall composite end point, death, appropriate implantable cardioverter-defibrillator (ICD) firing, atrial fibrillation, and heart failure (HF) with ejection fraction (EF) >55%. **C**, SARC+ vs SARC VUS: SARC+ and SARC VUS patients have similar risks except for a markedly higher hazard for left ventricular (LV) EF <35% and need for cardiac transplantation or ventricular assist device (VAD) in SARC+ patients. NYHA indicates New York Heart Association.

Survival analyses demonstrated that both SARC+ and SARC VUS patients had significantly earlier onset of events and a higher incidence of the overall composite outcome, HF, and AF than SARC− patients (Figure 3A, 3C, and 3D). SARC+ patients had significantly higher risk for the ventricular arrhythmia composite compared with SARC− patients, but no significant difference was seen between the SARC VUS and SARC− cohorts (Figure 3B). No significant difference was identified when each of the composite outcomes and AF were compared in the SARC+ and SARC VUS cohorts. Cox analyses demonstrated a higher incidence of adverse events in SARC+ versus SARC− patients across all composite and individual outcomes (Figure 4A). SARC+ patients had at least twice the lifetime hazard of death, HF, malignant arrhythmias, and AF compared with SARC− patients; risk for cardiac transplantation/LV assist device support was >4 times higher (hazard ratio [HR], 4.6; 95% CI, 2.3–9.4). Similarly, SARC VUS patients had a significantly higher incidence of the overall composite (HR, 1.5; 95% CI, 1.2–1.9), death (HR, 1.9; 95% CI, 1.1–3.4), and AF (HR, 1.8; 95% CI, 1.3–2.5) compared with SARC− patients (Figure 4B). In contrast, HRs were similar between SARC+ and SARC VUS patients, although SARC+ patients had a nearly 6-fold increase in risk for LV ejection fraction <35% (Figure 4C). Patients with >1 mutation (SARC2+) had the highest risk for transplantation/LV assist device (HR, 7.5; 95% CI, 2.7–20.5 compared with patients with 1 mutation) and stroke (HR, 5.1; 95% CI, 2.0–12.6; Figure III in the online-only Data Supplement). In a comparison of patients with mutations in *MYH7* and *MYBPC3, MYH7* mutation carriers had a ≈1.7- to nearly 3-fold higher risk of the overall composite outcome, AF, and advanced HF (New York Heart Association functional class III/IV and/or need for cardiac transplantation or LV assist device; Figure IV in the online-only Data Supplement).

### Predictors of Clinical Outcomes in the Genotyped HCM Cohort

Multivariable models were developed to identify patient characteristics associated with the composite outcomes and AF (Table 2). After controlling for proband status, sex, and race, the presence of a sarcomere mutation carried a >2-fold increased risk for all outcomes, highest for ventricular arrhythmias (HR, 2.8; 95% CI, 2.1–3.9; *P*<0.001). Because sarcomere status is correlated to age at diagnosis, models were also run including age at diagnosis as a covariate. The hazard for SARC+ was attenuated but remained significantly elevated after adjustment for age at diagnosis (data not shown), supporting that the increased hazard associated with carrying a sarcomere mutation is not mediated entirely by earlier age at diagnosis. Woman had an almost 30% lower risk for AF and malignant arrhythmias but a similarly higher risk for HF. Patients with multiple pathogenic or likely pathogenic sarcomere mutations had >2-fold increased risk for the overall composite and >4-fold increased risk for ventricular arrhythmias relative to patients without sarcomere mutations (Table III in the online-only Data Supplement).

**Table 2.**
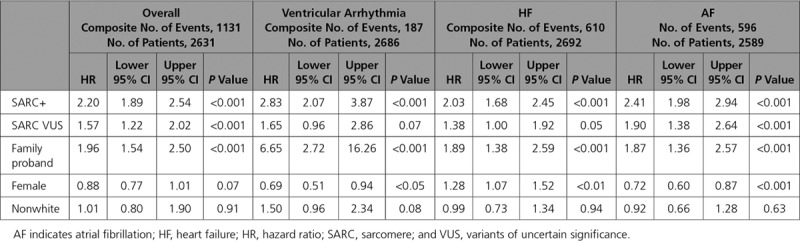
Multivariable Models Predicting Outcomes in the Genotyped Hypertrophic Cardiomyopathy Cohort

Patients with nonfamilial HCM (SARC− and no family history of HCM) had a lower risk of mortality and composite outcomes than other patients with HCM (HR 0.57 [0.49, 0.65] for the overall composite outcome and 0.46 [0.33, 0.64] for death). In addition, their age-specific mortality was similar to that of the US general population (Figure 2B).

## Discussion

SHaRe represents the largest comprehensive HCM cohort assembled to date, thereby providing critical new insights into natural history and lifetime risk of disease complications. In an examination of >24 000 patient-years of experience with disease, the cumulative burden of HCM was shown to be substantial, particularly for patients diagnosed earlier in life and those with sarcomere mutations. Although the incidence of ventricular arrhythmias declined with age, the risk for HF and AF increased, becoming most prevalent by late adulthood, even in patients diagnosed before 40 years of age. These observations underscore the need for lifelong surveillance and for developing effective strategies to prevent the progressive and adverse cardiac remodeling that leads to these complications.

### Morbidity and Mortality in HCM

Published HCM natural history studies reflect a median of ≈3700 patient-years of follow-up and have not considered cumulative risks of morbidity and mortality, nor have they systematically included genetic background.^2,6–11^ In contrast, we leveraged longitudinal data from a large international cohort to provide more precise and widely representative characterization of event rates and disease burden throughout life. Data from SHaRe demonstrate that mortality in patients with HCM is significantly higher than in the US general population. Although SHaRe data confirmed that absolute mortality in young patients with HCM is low, it was 4-fold higher than expected for 20- to 29-year-olds. In addition, although prior natural history studies (combined n=1700) reported sudden cardiac death as the leading cause of mortality in HCM, accounting for ≈40% of deaths,^2,6^ our data (n=4591) indicated that 16% of deaths were sudden. Overall mortality in SHaRe was driven by HF and noncardiac death, both of which were more common than lethal arrhythmias, consistent with recent evidence from other cohorts.^4^ Nonetheless, among patients diagnosed at a young age (<40 years), the cumulative incidence of lethal arrhythmias was 26% by 60 years of age, underscoring the need to incorporate age-specific risk predictions into clinical decision-making for primary prevention implantable cardioverter-defibrillator therapy. Further studies are needed to determine whether incorporating age at diagnosis, in addition to current age, improves sudden cardiac death risk assessment and predictive scores.^12^

Cumulative morbidity in HCM was dominated by complications related to HF and AF. Among patients diagnosed before 40 years of age, the risk of having HF or AF by age 60 years was 47% (95% CI, 42–52) and 62% (95% CI, 56–67), respectively. For context, a 40-year-old in the US general population has a lifetime risk of 20% to 45% for HF and 23% to 26% (women and men, respectively) for AF.^13^ SHaRe also demonstrated that regardless of age at diagnosis, the majority of HCM-related complications occurred later in life, peaking between 50 and 70 years of age. Therefore, continuing clinical surveillance in older patients with HCM is critical. Although ventricular arrhythmias and sudden death were indeed rare in those diagnosed at >60 years of age (2% cumulative incidence in SHaRe), all other risks, including AF, HF, and overall mortality, were greatest in this age group.

When incident onset of events after diagnosis was examined, patients diagnosed at an older age appeared to have more rapid occurrence of events compared with those diagnosed young. These observations suggest that although patients who are younger at diagnosis are more likely to experience at least 1 adverse event over the course of their lives, this burden is amortized over a long period of time. In contrast, patients who are older at diagnosis appear to have a lower cumulative risk of adverse events over their lifetime. However, they are more likely than younger patients to have an incident event on an annualized basis after diagnosis, presumably caused at least partially by being older and having more comorbidities at the time of diagnosis. Ultimately, most patients with HCM are likely to experience adverse events.

The observed lag time from HCM diagnosis to most severe outcomes, particularly in those diagnosed as children or young adults, implies that adverse remodeling progresses throughout life. Thus, there may be an extended window of opportunity to implement disease-modifying therapies. There is a critical need to better understand the factors driving disease progression and to develop effective treatment to delay or prevent the adverse remodeling that leads to HF and AF rather than treating these complications once they have already developed. To date, no such therapy is available for HCM. Translational approaches such as attenuation of fibrosis^14–16^ or allosteric myosin inhibitors^17^ may prove instrumental to hold disease in an early, relatively quiescent phase, thereby preventing adverse outcomes. Although these interventions may be predicted to have greatest impact in the preclinical phase of disease in young, at-risk mutation carriers,^14,18^ the possibility of interrupting disease progression even after full phenotypic development should also be actively pursued.

### Genotype Influences Outcome

Prior small cohort studies suggested an excess hazard and earlier presentation associated with sarcomere mutations.^8,9,19–21^ Data from this large multicenter cohort with rigorously curated genetic variants decisively support the conclusion that sarcomeric HCM is associated with worse outcomes than disease not caused by sarcomere mutations. Patients carrying sarcomere mutations with strong evidence for pathogenicity exhibited clinical disease at an earlier age and had a greater burden and earlier onset of HCM-related complications. The risk of developing the overall composite outcome by 50 years of age was 29% for SARC+ patients versus 14% for SARC− patients. Even after adjustment for earlier age at presentation, sarcomere mutations conferred significant excess hazard. HCM management guidelines from the American College of Cardiology Foundation/American Heart Association and European Society of Cardiology recommend genetic testing (Class IB) primarily to direct cascade assessments in family members. This study also underscores the value of genotype in guiding clinical management and determining the prognosis of patients with HCM. Indeed, our data suggest that mortality in patients with nonfamilial HCM (no sarcomere mutation and no family history of HCM) may not be significantly higher than that of the US general population. Although this finding requires confirmation and further characterization, it indicates that distinct disease subsets are currently grouped under the same broad designation of HCM, highlighting the need for more precise diagnostic strategies to improve risk stratification and clinical management.

Sarcomere variants of unknown significance identified in patients with HCM are typically disregarded because of the ambiguity surrounding their clinical relevance. However, our data indicate that these variants can affect prognosis. SARC VUS patients were diagnosed earlier and had worse outcomes than SARC− patients. Indeed, SHaRe data positioned outcome risk associated with SARC VUS HCM as intermediate between SARC+ and SARC−. These findings imply that some VUSs are pathogenic variants, even if they cannot be interpreted as such by current clinical variant classification strategies that require stringent evidence to allow confident use in family cascade screening or predictive genetic testing. Improved analytical pipelines and methods to determine variant pathogenicity are clearly needed to improve risk stratification and to enable predictive testing of family members.

### Limitations

Inherent limitations to retrospective, registry-based observational studies are survivor bias and the fact that inferences about causality cannot be made. Analyses of other large cohorts such as the Hypertrophic Cardiomyopathy Registry and the European HCM Registry^22–24^ will provide important opportunities for collaborative study and confirmation of key findings.

Our estimates of cumulative morbidity are subject to the ascertainment limitations of the current SHaRe database, including the retrospective data collection and lack of data on recurrent events. Given these limitations, cumulative morbidity from birth in the older age at diagnosis groups may be underestimated because these patients have a longer unobserved period when historical ascertainment of events may be less complete and a shorter period of follow-up after entry into SHaRe. Furthermore, cumulative incidence estimates at older ages in the age at diagnosis <40 years stratum should be interpreted cautiously because of the small numbers of patients remaining at risk at these later ages. Because of the detailed collection of medical history at entry into SHaRe, we feel that historical events have been reliably captured and are as complete as possible. Recurrent events are not yet available in the SHaRe database, but efforts are underway to incorporate recurrent events in future studies to more completely account for the cumulative burden of disease.

A limited subset of patients were potentially lost to follow-up, however the proportions were similar across a range of ages of diagnosis. Because baseline characteristics in this subset were similar to the overall cohort, systematic bias resulting from patients lost to follow-up is unlikely (Table IV in the online-only Data Supplement). We also acknowledge that appropriate implantable cardioverter-defibrillator therapy is an imperfect surrogate for sudden cardiac death and likely overestimates risk because not all treated episodes may have been fatal.^25^ This may particularly be the case for episodes treated only with antitachycardia pacing. Cox models for the ventricular arrhythmia composite did not differ significantly after the exclusion of appropriate implantable cardioverter-defibrillator therapy events made up of only antitachycardia pacing (18 of 104 events; Table V in the online-only Data Supplement). Finally, SHaRe represents primarily an adult-onset cohort; only 422 patients (9%) were <18 years old at the initial clinic visit. Efforts are underway to increase pediatric HCM representation and to compare pediatric with adult-onset disease.

## Conclusions

The cumulative morbidity and mortality of HCM are substantial and disproportionately borne by patients diagnosed earlier in life and patients with sarcomere mutations. Younger age at diagnosis and the presence of a sarcomere mutation were strong predictors of adverse events and should be incorporated into discussions about the prognosis for individual patients with HCM. Because the burden of HCM was dominated by HF and AF that developed later in life, continued surveillance in older patients, currently considered at low risk for HCM complications, is vital. Finally, a critical need and opportunity exist for developing disease-modifying therapies to interrupt progressive remodeling and adverse outcomes in HCM.

## Acknowledgments

Statistical analysis and expertise were additionally provided by Elyse Swallow Ivan Rybkin and Intekhab Hossain (Analysis Group, Boston, MA). The authors are grateful for the dedicated work of the site data managers: Hoshang Farhad, Efhalia Kaynor, and Kavitha Nutakki (Brigham and Women’s Hospital), Pieter Vriesendorp and Hannah van Velzen (Erasmus Medical Center), Fausto Barlocco (Careggi University Hospital), and Maryann Concannon (University of Michigan).

## Sources of Funding

Funding for SHaRe has been provided through an unrestricted research grant from Myokardia, Inc, a startup company that is developing therapeutics that target the sarcomere. MyoKardia, Inc, had no role in approving the content of this manuscript.

Dr Ho is supported by funding from the National Institutes of Health (1P50HL112349 and 1U01HL117006). Dr Day is supported by funding from the National Institutes of Health (R01 GRANT11572784), the American Heart Association (grant in aid), and the Taubman Medical Institute (University of Michigan). Dr Olivotto, Dr Cecchi, and F. Girolami are supported by the Italian Ministry of Health (“Left Ventricular Hypertrophy in Aortic Valve Disease and Hypertrophic Cardiomyopathy: Genetic Basis, Biophysical Correlates and Viral Therapy Models” [RF-2013-02356787] and NET-2011-02347173 [Mechanisms and Treatment of Coronary Microvascular Dysfunction in Patients With Genetic or Secondary Left Ventricular Hypertrophy]) and by the Tuscany Registry of Sudden Cardiac Death (ToRSADE) project (FAS-Salute 2014, Regione Toscana). Dr Ware is supported by the Wellcome Trust (107469/Z/15/Z) and the Medical Research Council (United Kingdom). Dr Seidman is funded by the Howard Hughes Medical Institute.

## Disclosures

Drs Ho, Day, Olivotto, Colan, and Ashley receive research support from MyoKardia, Inc. Drs Seidman, Green, and Fox own shares in Myokardia, Inc (Dr Seidman is also a founder). The other authors report no conflicts.

## Supplementary Material
