# Supplementary material for: Genotype and Lifetime Burden of Disease in Hypertrophic Cardiomyopathy: Insights From the Sarcomeric Human Cardiomyopathy Registry (SHaRe)
Source: Circulation. 2018 Oct 1;138(14):1387–98. doi: 10.1161/CIRCULATIONAHA.117.033200 (PMC6170149; doi:10.1161/CIRCULATIONAHA.117.033200)
Supplement: Supplementary file 1 [file cir-138-1387-s001.pdf]

SUPPLEMENTAL METHODS

Classification of Genetic Variants

In addition to the information and interpretation provided by the genetic testing laboratory, all variants underwent additional scrutiny in the SHaRe database through systematic review by a subgroup of SHaRe investigators (A.L.C, S.M.D, J.S.W, C.Y.H). Population allele frequency was assessed in ExAC[1] using the method described in Whiffin et al,[2] with $4x10^{-5}$ as the maximum credible population allele frequency for a causative variant. All rare truncating variants in *MYBPC3* were considered pathogenic since loss of function in this gene is a well-established disease mechanism.[3] Missense *MYBPC3* variants classified as likely pathogenic or pathogenic by individual sites were reviewed, considering segregation data and ExAC frequency. If segregation criteria were not met (<5 meioses) and a variant was not enriched in disease versus control populations, then the variant was reclassified as VUS (if rare in ExAC) or likely benign (if above frequency threshold in EXAC) in SHaRe. Variants with discordant classifications across SHaRe sites were resolved using the same criteria to designate a single SHaRe classification.

Statistical Methods

<u>Cumulative incidence from birth, stratified by age at diagnosis</u>

To examine whether patients' age-specific disease burden varies based on their age at HCM diagnosis, the cumulative incidences of key outcomes over the period from birth until the end of follow-up were estimated separately in each of four strata of age of diagnosis (<40 to 60, 60+ years).
Kaplan-Meier curves were fit separately in each of the three age of diagnosis strata for four key outcomes (time from birth to occurrence of the overall composite, ventricular arrhythmia composite, heart failure composite, and atrial fibrillation). The cumulative incidence for each outcome was estimated as 1 minus the survivor function in each stratum of diagnosis age.

<u>Comparison of age-specific mortality in US HCM cohort versus US general population</u>

Mortality was estimated in these two populations for each of the following ten-year age intervals: 20-29, 30-39, 40-49, 50-59, and 60-69 years. In the full HCM cohort, mortality in these 10-year intervals was estimated from the Kaplan-Meier analyses of mortality. In particular, mortality within a specific ten-year age interval was estimated by taking the difference between the cumulative incidence estimates obtained at the upper and lower limits of that age interval. Confidence intervals were obtained by taking the 2.5^{th} and 97.5^{th} percentiles of the age-specific risks estimated from 500 bootstrap samples.
Age-specific mortality in the US general population was estimated using the CDC Wonder database (http://wonder.cdc.gov). This database provides estimates of deaths and population counts in 5-year age intervals (e.g., 20-24, 25-29) based on US population and mortality data from 1999-2014; estimates of deaths and population counts over ten-year age intervals (e.g., 20-29) can be obtained by summing the reported quantities across the constituent 5-year age intervals. Mortality in each ten-year age interval was then estimated as the number of deaths divided by the total population count in that interval. In each age interval, a z-test of the difference between proportions was used to compare the mortality estimates in these two populations. For the z-test, standard errors in the US general population cohort were calculated directly from the raw data, while standard errors in the HCM cohort were estimated from the 95% confidence intervals obtained from bootstrapping.

<u>Regression models</u>

In the genotyped cohort, the association between sarcomere status (defined as SARC +, SARC VUS and SARC -) and key outcomes (overall composite, ventricular arrhythmia composite, heart failure composite and atrial fibrillation) was also investigated in multivariable models. Time to event was measured from

birth in these models. Independent variables included proband status (yes versus no), sex (female versus male), race (white versus non-white) and age at HCM diagnosis (<40 versus ≥ 40). Additional models that separated out SARC + patients into those with at least 2 pathogenic/likely pathogenic mutations (or 1 pathogenic/likely pathogenic and at least 1 VUS mutation), and examined the risk in this subgroup in relation to the other sarcomere status subgroups were also fit.

Annualized incidence of events following HCM diagnosis

Occurrence of incident events following HCM diagnosis was investigated for the overall composite outcome, in the overall population, and by age of diagnosis strata. These rates were calculated among patients without a history of the event at the time of diagnosis. In particular, the annualized risk in each group was calculated by dividing the proportion of patients in each group who had the event by the mean follow-up time in years of each group.

Advanced heart failure stratified by left ventricular ejection fraction (LVEF)

The burden of advanced heart failure (defined as NYHA functional class III-IV and/or need for cardiac transplantation or left ventricular assist device) was investigated in the subgroup of patients who had an echo within 1 year of their NYHA III/IV event, or end of follow-up. Heart failure patients were stratified according to LVEF <55% or ≥55%. Cumulative incidences of heart failure with EF < 55% and ≥55% in the full HCM cohort were estimated from Kaplan-Meier analyses of these outcomes. In the genotyped cohort, separate Kaplan-Meier analyses and Cox models were fit to examine the association between sarcomere status and time from birth to development of heart failure.

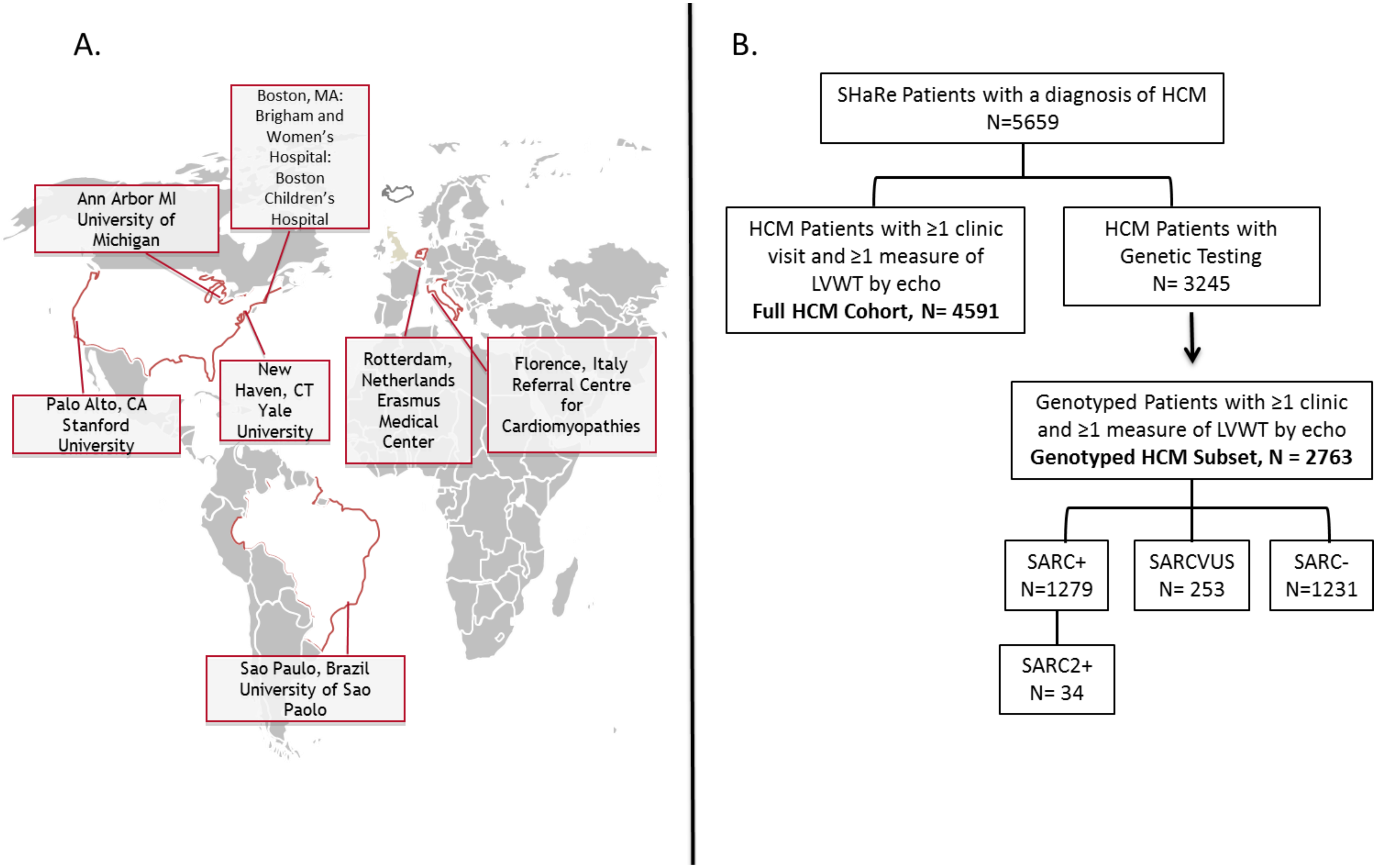

Supplemental Figure 1. Map depicting participating sites in SHaRe and overview of selection of subjects for this study

A. SHaRe Sites and country of location are shown on a global map

B. Study Subject Selection Flow Diagram

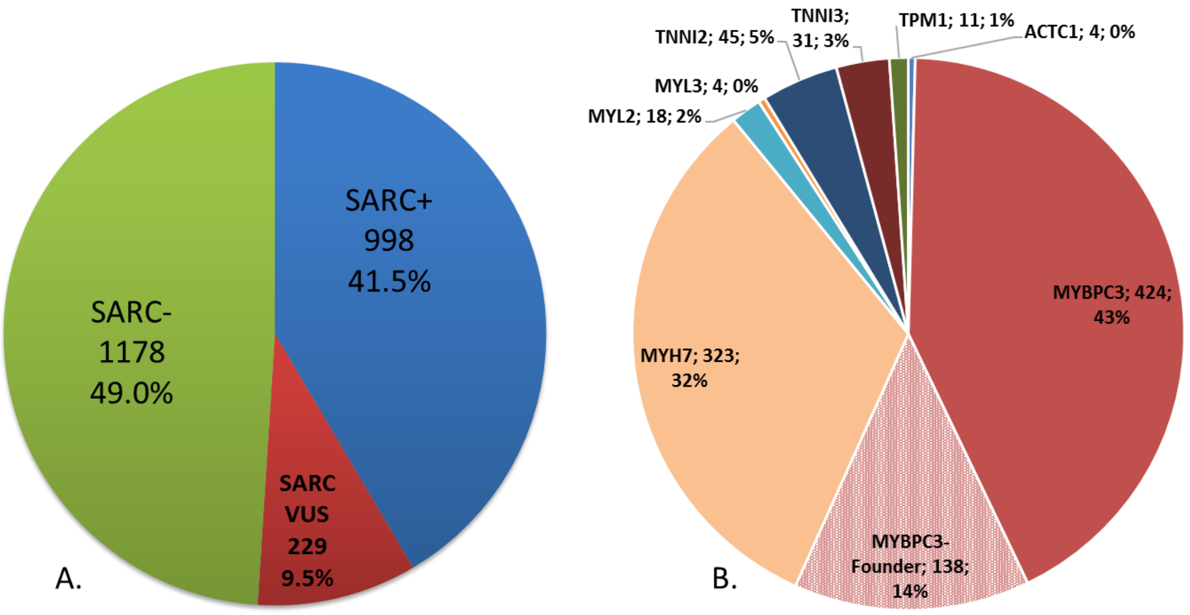

Supplemental Figure 2. Genetic testing results in 2405 HCM probands

A. Of HCM probands who underwent genetic testing, 41.5% had a pathogenic or likely pathogenic sarcomere mutation identified, 9.5% had a sarcomere gene variant of unknown significance, and 49.0% had no sarcomere variant identified.
B. In the SARC + cohort, variants were most commonly identified in *MYBPC3* (57%, including 14% with founder mutations) and *MYH7* (*32*%)

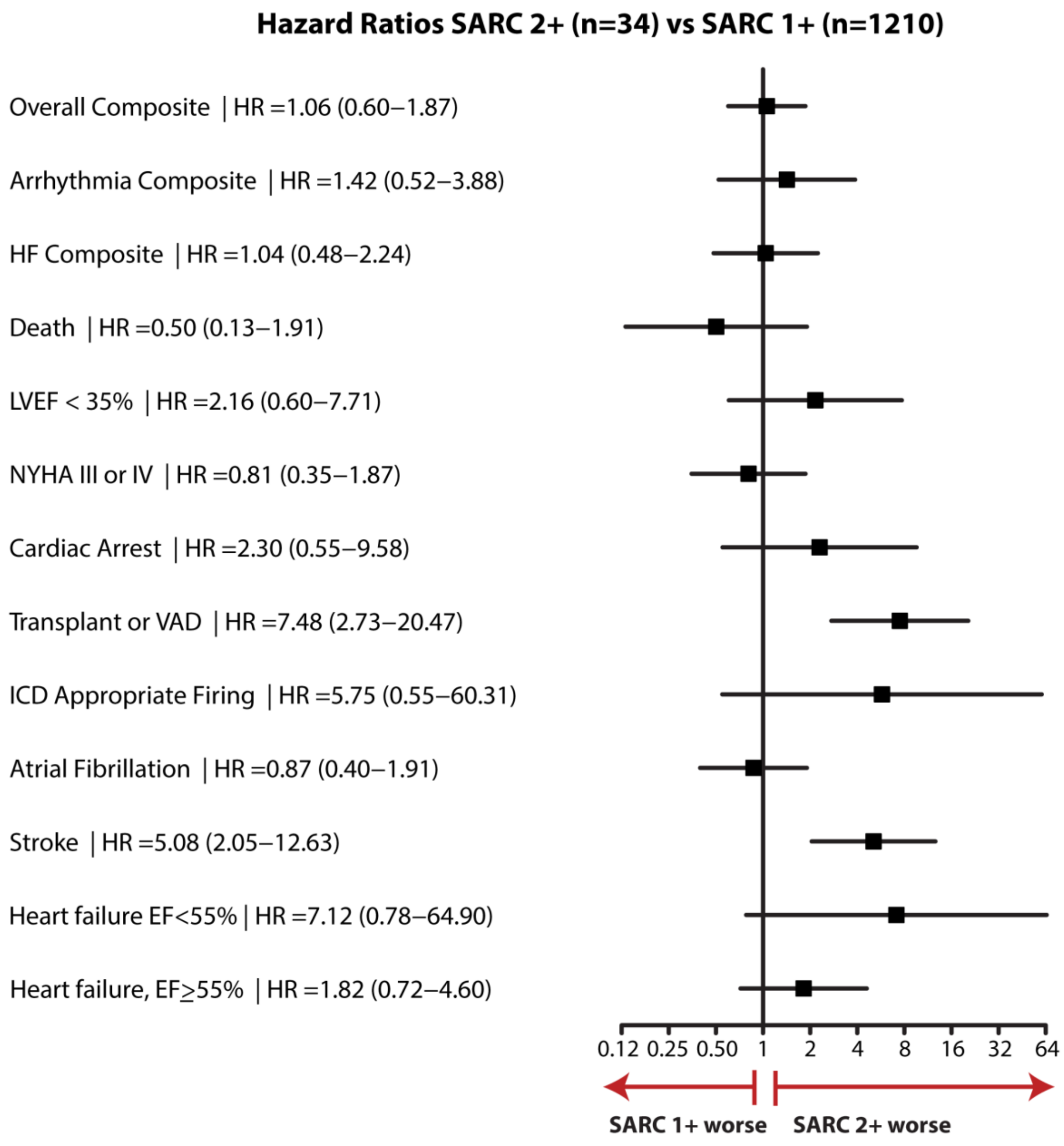

Supplemental Figure 3. Forest plot comparing outcomes in HCM patients carrying multiple (SARC2+) versus single (SARC1+) pathogenic or likely pathogenic sarcomere variants. SARC2+ patients have substantially increased risk of stroke, heart failure with reduced ejection fraction and need for cardiac transplantation or left ventricular assist device.

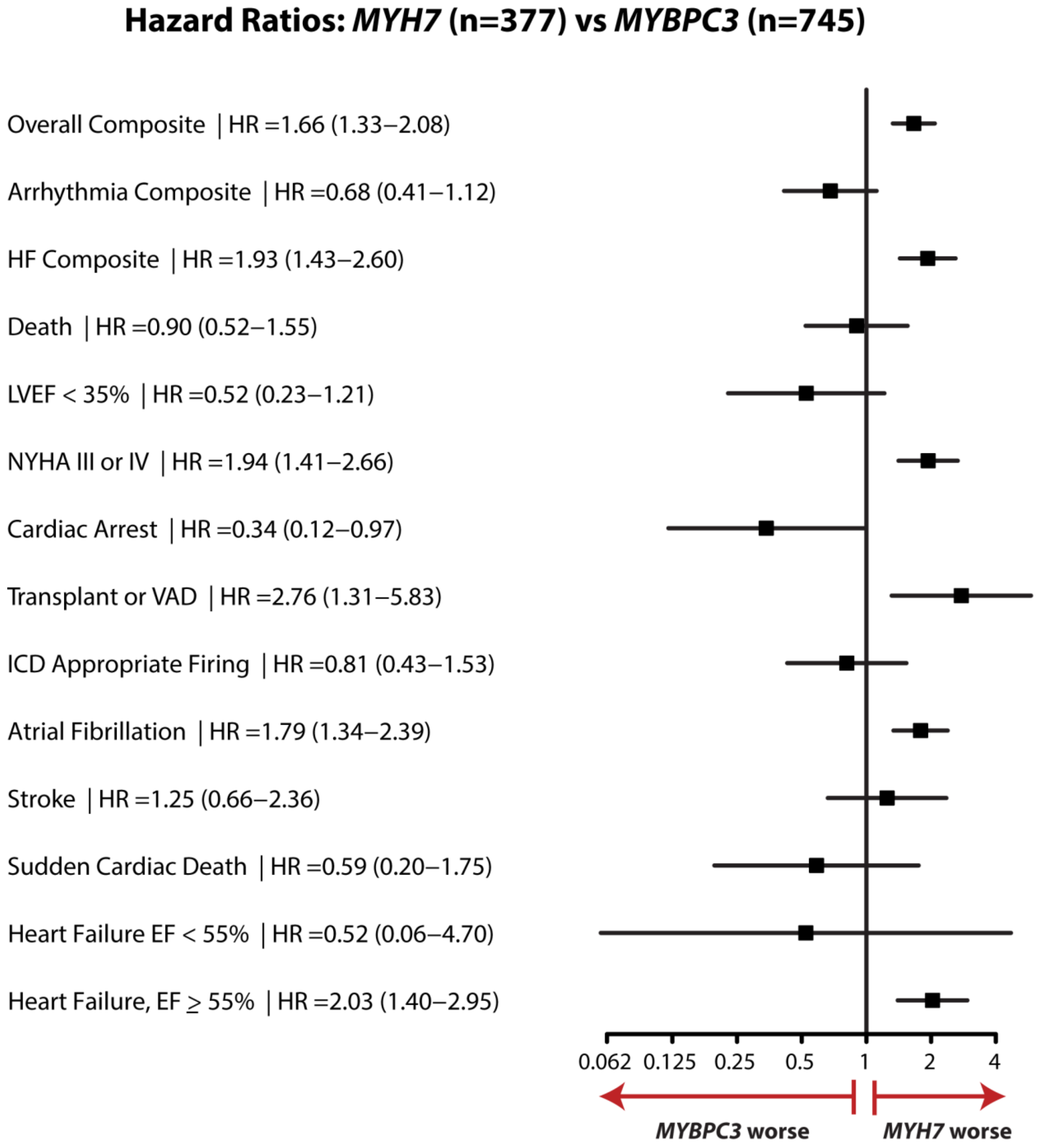

Supplemental Figure 4. Forest plot comparing outcomes in *MYBPC3* and *MYH7* mutation carriers. Patients with HCM caused by mutations in *MYH7* have worse outcomes with approximately 2-fold increased risk for the overall and heart failure composites, need for transplantation/LVAD, and atrial fibrillation.

Supplemental Table 1. Sarcomere gene variants identified in the SHaRe genotyped cohort

Gene	c. designation	p. designation	Classification	Count
**ACTC1**	c.268C>T	p.His90Tyr	VUS	1
**ACTC1**	c.275_277del	p.Phe92del	P/LP	1
**ACTC1**	c.281A>G	p.Asn94Ser	P/LP	1
**ACTC1**	c.301G>A	p.Glu101Lys	P/LP	1
**ACTC1**	c.514G>A	p.Ala172Thr	VUS	1
**ACTC1**	c.695C>T	p.Ala232Val	P/LP	2
**ACTC1**	c.818C>T	p.Ser273Phe	VUS	1
**ACTC1**	c.854T>C	p.Met285Thr	VUS	1
**MYBPC3**	c.3G>C	p.Met1?	P/LP	2
**MYBPC3**	c.26-2A>G		P/LP	2
**MYBPC3**	c.65_66insG	p.Ala23Argfs*26	P/LP	1
**MYBPC3**	c.95del	p.Glu32Glyfs*7	P/LP	1
**MYBPC3**	c.104G>A	p.Arg35Gln	VUS	1
**MYBPC3**	c.177_187del	p.Glu60fs*49	P/LP	2
**MYBPC3**	c.208del	p.Glu70fs*26	P/LP	1
**MYBPC3**	c.223G>A	p.Asp75Asn	VUS	1
**MYBPC3**	c.227_228insA	p.Ser78Ilefs*35	P/LP	1
**MYBPC3**	c.237C>G	p.Tyr79*	P/LP	1
**MYBPC3**	c.292G>C	p.Glu98Gln	VUS	1
**MYBPC3**	c.306_306del	p.Met103Cysfs*56	P/LP	1
**MYBPC3**	c.340A>G	p.Thr114Ala	VUS	1
**MYBPC3**	c.362del	p.Pro121Argfs*38	P/LP	1
**MYBPC3**	c.373G>T	p.Ala125Ser	VUS	1
**MYBPC3**	c.407-1G>A		P/LP	1
**MYBPC3**	c.410C>G	p.Ser137*	P/LP	1
**MYBPC3**	c.436_437insA	p.Thr146Asnfs*7	P/LP	1
**MYBPC3**	c.442G>A	p.Gly148Arg	VUS	3
**MYBPC3**	c.459del	p.Ile154Leufs*5	P/LP	2
**MYBPC3**	c.471C>T	p.Phe157=	VUS	1
**MYBPC3**	c.481C>T	p.Pro161Ser	P/LP	8
**MYBPC3**	c.495G>C	p.Glu165Asp	VUS	1
**MYBPC3**	c.497T>G	p.Val166Gly	VUS	1
**MYBPC3**	c.506-1G>A		P/LP	1
**MYBPC3**	c.506-2A>C		P/LP	4
**MYBPC3**	c.518C>A	p.Thr173Asn	VUS	1
**MYBPC3**	c.540_559del	p.Ala181Cysfs*53	P/LP	1
**MYBPC3**	c.551dup	p.Lys185Glufs*56	P/LP	2

**MYBPC3**	c.636C>G	p.Ser212Arg	VUS	2
**MYBPC3**	c.649A>C	p.Ser217Arg	VUS	1
**MYBPC3**	c.654+1G>A		P/LP	5
**MYBPC3**	c.655G>C†	p.Val219Leu	P/LP	1
**MYBPC3**	c.676_701dup	p.Gly235Serfs*74	P/LP	1
**MYBPC3**	c.710A>C	p.Tyr237Ser	P/LP	1
**MYBPC3**	c.756C>A	p.Phe252Leu	VUS	1
**MYBPC3**	c.772+1 G>A		P/LP	1
**MYBPC3**	c.772G>A†	p.Glu258Lys	P/LP	125
**MYBPC3**	c.818G>A	p.Arg273His	VUS	1
**MYBPC3**	c.821+1G>A		P/LP	9
**MYBPC3**	c.884T>C	p.Phe295Ser	VUS	1
**MYBPC3**	c.897del	p.Lys301Argfs*49	P/LP	2
**MYBPC3**	c.906-36G>A		VUS	1
**MYBPC3**	c.913_914del	p.Phe305Profs*27	P/LP	13
**MYBPC3**	c.913del	p.Phe305Profs*27	P/LP	1
**MYBPC3**	c.927-10C>A		P/LP	1
**MYBPC3**	c.927-2A>G		P/LP	174
**MYBPC3**	c.927-9G>A		P/LP	17
**MYBPC3**	c.932C>A	p.Ser311*	P/LP	1
**MYBPC3**	c.964T>C	p.Trp322Arg	VUS	1
**MYBPC3**	c.989del	p.Pro330Hisfs*20	P/LP	1
**MYBPC3**	c.993_994insT	p.Glu332*	P/LP	1
**MYBPC3**	c.1000G>T	p.Glu334*	P/LP	2
**MYBPC3**	c.1015C>T	p.Gln339*	P/LP	1
**MYBPC3**	c.1020C>G	p.Tyr340*	P/LP	6
**MYBPC3**	c.1053_1054delinsTT	p.Leu352Phefs*5	P/LP	2
**MYBPC3**	c.1069C>T	p.Arg357Cys	VUS	1
**MYBPC3**	c.1090G>A†	p.Ala364Thr	P/LP	4
**MYBPC3**	c.1112C>G	p.Pro371Arg	VUS	5
**MYBPC3**	c.1168del	p.His390Metfs*16	P/LP	1
**MYBPC3**	c.1171del	p.Asp391Thrfs*15	P/LP	1
**MYBPC3**	c.1174del	p.Ala392Leufs*14	P/LP	11
**MYBPC3**	c.1210C>T	p.Gln404*	P/LP	3
**MYBPC3**	c.1224-2A>G		P/LP	2
**MYBPC3**	c.1227-13G>A		P/LP	1
**MYBPC3**	c.1227-2A>G		P/LP	1
**MYBPC3**	c.1235_1236del	p.Phe412*	P/LP	1
**MYBPC3**	c.1238A>G	p.Glu413Gly	VUS	1
**MYBPC3**	c.1247G>T	p.Gly416Val	VUS	1
**MYBPC3**	c.1351+2T>C		P/LP	1

**MYBPC3**	c.1354_1355insC	p.Val454Cysfs*21	P/LP	1
**MYBPC3**	c.1404del	p.Gln469Serfs*19	P/LP	1
**MYBPC3**	c.1408C>T	p.Arg470Trp	VUS	1
**MYBPC3**	c.1456T>G	p.Trp486Gly	VUS	1
**MYBPC3**	c.1458-1G>A		P/LP	8
**MYBPC3**	c.1458-1G>C		P/LP	8
**MYBPC3**	c.1483C>G	p.Arg495Gly	P/LP	1
**MYBPC3**	c.1483C>T	p.Arg495Trp	P/LP	2
**MYBPC3**	c.1484G>A	p.Arg495Gln	P/LP	19
**MYBPC3**	c.1504C>T	p.Arg502Trp	P/LP	16
**MYBPC3**	c.1505G>A	p.Arg502Gln	P/LP	29
**MYBPC3**	c.1509C>G	p.Phe503Leu	P/LP	2
**MYBPC3**	c.1513_1515del	p.Lys505del	VUS	1
**MYBPC3**	c.1548-1G>A		P/LP	1
**MYBPC3**	c.1564G>A	p.Ala522Thr	VUS	1
**MYBPC3**	c.1565C>T	p.Ala522Val	VUS	1
**MYBPC3**	c.1568G>T	p.Gly523Val	VUS	2
**MYBPC3**	c.1574A>C	p.Tyr525Ser	VUS	1
**MYBPC3**	c.1575T>G	p.Tyr525*	P/LP	2
**MYBPC3**	c.1591G>C	p.Gly531Arg	VUS	7
**MYBPC3**	c.1624+1G>A		P/LP	5
**MYBPC3**	c.1624+4A>T		P/LP	8
**MYBPC3**	c.1624G>C†	p.Glu542Gln	P/LP	16
**MYBPC3**	c.1639del	p.Val547Cysfs*8	P/LP	4
**MYBPC3**	c.1684G>A	p.Ala562Thr	VUS	1
**MYBPC3**	c.1687del	p.Val563Cysfs*16	P/LP	1
**MYBPC3**	c.1696T>C	p.Cys566Arg	P/LP	2
**MYBPC3**	c.1720C>T	p.Arg574Trp	VUS	1
**MYBPC3**	c.1765C>G	p.Arg589Gly	VUS	5
**MYBPC3**	c.1766G>A	p.Arg589His	VUS	1
**MYBPC3**	c.1783A>G	p.Ile595Val	VUS	1
**MYBPC3**	c.1789C>T	p.Arg597Trp	VUS	2
**MYBPC3**	c.1790G>A	p.Arg597Gln	VUS	2
**MYBPC3**	c.1791-1G>A		P/LP	1
**MYBPC3**	c.1800del	p.Lys600Asnfs*2	P/LP	2
**MYBPC3**	c.1809T>G	p.Ile603Met	VUS	2
**MYBPC3**	c.1828G>A	p.Asp610Asn	VUS	1
**MYBPC3**	c.1828G>C	p.Asp610His	VUS	4
**MYBPC3**	c.1831G>A	p.Glu611Lys	VUS	3
**MYBPC3**	c.1838dup	p.Asp613Glufs*25	P/LP	1
**MYBPC3**	c.1892delT	p.Phe631Serfs*32	P/LP	3

**MYBPC3**	c.1895delT	p.Met632Argfs*31	P/LP	5
**MYBPC3**	c.1897+5G>A		VUS	1
**MYBPC3**	c.1898-1G>A		P/LP	1
**MYBPC3**	c.1900del	p.Val634Serfs*29	P/LP	1
**MYBPC3**	c.1928-2A>G		P/LP	14
**MYBPC3**	c.1999_2000del	p.Leu667Thrfs*25	P/LP	1
**MYBPC3**	c.1999_2000delCinsG	p.Leu667Aspfs*15	P/LP	1
**MYBPC3**	c.2025_2026del	p.Asp676Profs*16	P/LP	1
**MYBPC3**	c.2030C>T	p.Pro677Leu	VUS	1
**MYBPC3**	c.2048G>A	p.Trp683*	P/LP	1
**MYBPC3**	c.2056G>C	p.Ala686Pro	VUS	1
**MYBPC3**	c.2065C>T	p.Gln689*	P/LP	1
**MYBPC3**	c.2077G>T	p.Ala693Ser	VUS	1
**MYBPC3**	c.2096del	p.Pro699Glnfs*55	P/LP	1
**MYBPC3**	c.2113dup	p.Thr705Asnfs*3	P/LP	2
**MYBPC3**	c.2132G>A	p.Trp711*	P/LP	1
**MYBPC3**	c.2149-1G>A		P/LP	1
**MYBPC3**	c.2149-2del		P/LP	1
**MYBPC3**	c.2149-9C>A		P/LP	5
**MYBPC3**	c.2157_2158del	p.Cys719*	P/LP	1
**MYBPC3**	c.2182G>T	p.Glu728*	P/LP	1
**MYBPC3**	c.2210C>T	p.Thr737Met	VUS	1
**MYBPC3**	c.2221del	p.Ala741Glnfs*13	P/LP	1
**MYBPC3**	c.2258dup	p.Lys754Glufs*79	P/LP	7
**MYBPC3**	c.2267del	p.Pro756Leufs*66	P/LP	1
**MYBPC3**	c.2308+1G>A		P/LP	2
**MYBPC3**	c.2308+1G>T		P/LP	2
**MYBPC3**	c.2308+2T>G		P/LP	1
**MYBPC3**	c.2308G>A†	p.Asp770Asn	P/LP	6
**MYBPC3**	c.2309-2A>G		P/LP	8
**MYBPC3**	c.2311G>A	p.Val771Met	VUS	2
**MYBPC3**	c.2324C>G	p.Pro775Arg	VUS	1
**MYBPC3**	c.2356G>T	p.Asp786Tyr	VUS	1
**MYBPC3**	c.2371C>T	p.Gln791*	P/LP	1
**MYBPC3**	c.2373insG	p.Trp792Valfs*41	P/LP	96
**MYBPC3**	c.2374dup	p.Trp792Valfs*41	P/LP	1
**MYBPC3**	c.2374T>C	p.Trp792Arg	P/LP	10
**MYBPC3**	c.2376G>A	p.Trp792*	P/LP	4
**MYBPC3**	c.2391C>A	p.Tyr797*	P/LP	2
**MYBPC3**	c.2394_2395insT	p.Gly799Trpfs*34	P/LP	1
**MYBPC3**	c.2398G>A	p.Gly800Arg	VUS	1

**MYBPC3**	c.2414-1G>A		P/LP	1
**MYBPC3**	c.2414-2A>G		P/LP	1
**MYBPC3**	c.2429G>A	p.Arg810His	P/LP	13
**MYBPC3**	c.2429G>T	p.Arg810Leu	P/LP	5
**MYBPC3**	c.2431_2433del	p.Lys811del	VUS	1
**MYBPC3**	c.2432A>G	p.Lys811Arg	VUS	1
**MYBPC3**	c.2435A>G	p.Lys812Arg	VUS	1
**MYBPC3**	c.2454G>A	p.Trp818*	P/LP	4
**MYBPC3**	c.2458C>T	p.Arg820Trp	VUS	1
**MYBPC3**	c.2459G>A	p.Arg820Gln	VUS	1
**MYBPC3**	c.2490dup	p.His831Serfs*2	P/LP	1
**MYBPC3**	c.2526C>G	p.Tyr842*	P/LP	1
**MYBPC3**	c.2541C>G	p.Tyr847*	P/LP	2
**MYBPC3**	c.2543_2544dup	p.Val849Argfs*31	P/LP	1
**MYBPC3**	c.2543C>G	p.Ala848Gly	VUS	1
**MYBPC3**	c.2550C>G	p.Asn850Lys	VUS	1
**MYBPC3**	c.2550del	p.Asn850Lysfs*29	P/LP	2
**MYBPC3**	c.2555dup	p.Glu853Argfs*31	P/LP	2
**MYBPC3**	c.2558G>T	p.Gly853Val	VUS	1
**MYBPC3**	c.2573G>A	p.Ser858Asn	VUS	3
**MYBPC3**	c.2603-1G>A		P/LP	1
**MYBPC3**	c.2670_2671insG	p.Arg891Alafs*160	P/LP	3
**MYBPC3**	c.2670G>A	p.Trp890*	P/LP	7
**MYBPC3**	c.2672G>A	p.Arg891Gln	VUS	1
**MYBPC3**	c.2689_2698del	p.Gly897Alafs*24	P/LP	6
**MYBPC3**	c.2710del	p.Tyr904Thrfs*20	P/LP	4
**MYBPC3**	c.2715C>A	p.Ser905Arg	VUS	1
**MYBPC3**	c.2737+2T>A		P/LP	1
**MYBPC3**	c.2737+5G>A		P/LP	1
**MYBPC3**	c.2747G>A	p.Trp916*	P/LP	3
**MYBPC3**	c.2792dup	p.Lys932Glufs*119	P/LP	1
**MYBPC3**	c.2815C>T	p.Arg939Trp	VUS	1
**MYBPC3**	c.2827C>T	p.Arg943*	P/LP	50
**MYBPC3**	c.2833_2834del	p.Arg945Glyfs*105	P/LP	1
**MYBPC3**	c.2834G>A	p.Arg945Leu	VUS	1
**MYBPC3**	c.2839C>A	p.His947Asn	VUS	1
**MYBPC3**	c.2849C>T	p.Ala950Val	VUS	1
**MYBPC3**	c.2862_2865del	p.Pro955Leufs*7	P/LP	2
**MYBPC3**	c.2864_2865del	p.Pro955Argfs*95	P/LP	41
**MYBPC3**	c.2869dup	p.Thr957fs*94	P/LP	1
**MYBPC3**	c.2893C>T	p.Gln965*	P/LP	1

**MYBPC3**	c.2905+1G>A		P/LP	8
**MYBPC3**	c.2905C>T	p.Gln969*	P/LP	3
**MYBPC3**	c.2908C>T	p.Arg970Trp	VUS	1
**MYBPC3**	c.2909G>A	p.Arg970Gln	VUS	1
**MYBPC3**	c.2943_2947delGACCA	p.Gln981Hisfs*68	P/LP	1
**MYBPC3**	c.2992C>T	p.Gln998*	P/LP	1
**MYBPC3**	c.2994+2T>C		P/LP	2
**MYBPC3**	c.3005G>A	p.Arg1002Gln	VUS	1
**MYBPC3**	c.3019T>C	p.Trp1007Arg	VUS	1
**MYBPC3**	c.3029del	p.Glu1010Glyfs*10	P/LP	1
**MYBPC3**	c.3034C>T	p.Gln1012*	P/LP	1
**MYBPC3**	c.3040del	p.Leu1014Trpfs*6	P/LP	1
**MYBPC3**	c.3064C>T	p.Arg1022Cys	VUS	2
**MYBPC3**	c.3065G>A	p.Arg1022His	VUS	1
**MYBPC3**	c.3065G>C	p.Arg1022Pro	VUS	3
**MYBPC3**	c.3098G>A	p.Arg1033Gln	VUS	1
**MYBPC3**	c.3100del	p.Ala1034Profs*12	P/LP	1
**MYBPC3**	c.3127dup	p.Tyr1043Leufs*8	P/LP	1
**MYBPC3**	c.3129C>A	p.Tyr1043*	P/LP	1
**MYBPC3**	c.3181C>T	p.Gln1061*	P/LP	1
**MYBPC3**	c.3190+1G>A		P/LP	4
**MYBPC3**	c.3190+5G>A		P/LP	2
**MYBPC3**	c.3192dup	p.Lys1065Glnfs*12	P/LP	29
**MYBPC3**	c.3217dup	p.Arg1073Profs*4	P/LP	1
**MYBPC3**	c.3218G>C	p.Arg1073Pro	VUS	1
**MYBPC3**	c.3226_3227insT	p.Asp1076Valfs*6	P/LP	1
**MYBPC3**	c.3233G>A	p.Trp1078*	P/LP	4
**MYBPC3**	c.3277G>T	p.Gly1093Cys	VUS	1
**MYBPC3**	c.3284C>T	p.Thr1095Met	VUS	1
**MYBPC3**	c.3286G>T	p.Glu1096*	P/LP	4
**MYBPC3**	c.3288del	p.Glu1096Aspfs*93	P/LP	1
**MYBPC3**	c.3293G>A	p.Trp1098*	P/LP	1
**MYBPC3**	c.3297dup	p.Tyr1100Valfs*49	P/LP	1
**MYBPC3**	c.3300C>A	p.Tyr1100*	P/LP	1
**MYBPC3**	c.3330+2T>C		P/LP	2
**MYBPC3**	c.3330+2T>G		P/LP	9
**MYBPC3**	c.3330+5G>A		P/LP	2
**MYBPC3**	c.3331-1G>A		P/LP	2
**MYBPC3**	c.3331-1G>C		P/LP	1
**MYBPC3**	c.3331-2A>G		P/LP	1
**MYBPC3**	c.3332_3335dup4	p.Trp1112*	P/LP	1

**MYBPC3**	c.3334dup	p.Trp1112Leufs*37	P/LP	1
**MYBPC3**	c.3335G>A	p.Trp1112*	P/LP	1
**MYBPC3**	c.3340_3342del	p.Thr1114del	VUS	1
**MYBPC3**	c.3350+5G>C		P/LP	1
**MYBPC3**	c.3370T>C	p.Cys1124Arg	VUS	2
**MYBPC3**	c.3372C>A	p.Cys1124*	P/LP	2
**MYBPC3**	c.3407_3409del	p.Tyr1136del	VUS	3
**MYBPC3**	c.3413G>C	p.Arg1138Pro	VUS	2
**MYBPC3**	c.3414_3415insC	p.Val1139Argfs*10	P/LP	1
**MYBPC3**	c.3414dup	p.Val1139Argfs*10	P/LP	1
**MYBPC3**	c.3432_3435dup	p.Phe1147Trpfs*3	P/LP	2
**MYBPC3**	c.3452C>T	p.Ala1151Val	VUS	2
**MYBPC3**	c.3467dup	p.Pro1157Alafs*12	P/LP	1
**MYBPC3**	c.3480C>G	p.Ile1160Met	VUS	1
**MYBPC3**	c.3490+1G>A		P/LP	1
**MYBPC3**	c.3490+1G>T		P/LP	2
**MYBPC3**	c.3490+6G>A		VUS	1
**MYBPC3**	c.3514_3517dup	p.Lys1173Ilefs*2	P/LP	1
**MYBPC3**	c.3551C>A	p.Thr1184Asn	VUS	2
**MYBPC3**	c.3560T>G	p.Leu1187Arg	VUS	2
**MYBPC3**	c.3584G>T	p.Gly1195Val	VUS	1
**MYBPC3**	c.3599T>C	p.Leu1200Pro	VUS	1
**MYBPC3**	c.3614G>A	p.Arg1205Gln	VUS	1
**MYBPC3**	c.3617_3618del	p.Gly1206Glufs*35	P/LP	1
**MYBPC3**	c.3617del	p.Gly1206Valfs*31	P/LP	2
**MYBPC3**	c.3624_3625insC	p.Lys1209Glnfs*33	P/LP	4
**MYBPC3**	c.3624del	p.Lys1209Glnfs*28	P/LP	4
**MYBPC3**	c.3627+1G>A		P/LP	1
**MYBPC3**	c.3628-1G>A		P/LP	1
**MYBPC3**	c.3628-41_3628-17del		P/LP	4
**MYBPC3**	c.3640T>A	p.Trp1214Arg	VUS	1
**MYBPC3**	c.3642G>A	p.Trp1214*	P/LP	1
**MYBPC3**	c.3662_3662del	p.Leu1221Argfs*16	P/LP	6
**MYBPC3**	c.3690_3691del	p.Phe1230Leufs*11	P/LP	1
**MYBPC3**	c.3694A>T	p.Lys1232*	P/LP	1
**MYBPC3**	c.3697C>T	p.Gln1233*	P/LP	10
**MYBPC3**	c.3742_3759dup	p.Gly1248_Cys1253dup	P/LP	6
**MYBPC3**	c.3751T>C	p.Tyr1251His	VUS	2
**MYBPC3**	c.3767_3768del	p.Thr1256Lysfs*9	P/LP	2
**MYBPC3**	c.3767_3769del	p.Thr1256del	VUS	1
**MYBPC3**	c.3771C>A	p.Asn1257Lys	P/LP	1

**MYBPC3**	c.3776del	p.Gln1259Argfs*72	P/LP	1
**MYBPC3**	c.3776delA	p.Gln1259Argfs*72	P/LP	8
**MYBPC3**	c.3791G>T	p.Cys1264Phe	VUS	1
**MYBPC3**	c.3797G>A	p.Cys1266Tyr	VUS	1
**MYBPC3**	c.3808G>A	p.Val1270Met	VUS	1
**MYBPC3**	c.3811C>T	p.Arg1271*	P/LP	2
**MYBPC3**	c.3812G>A	p.Arg1271Gln	VUS	1
**MYBPC3**	c.3814+1G>A		P/LP	1
**MYBPC3**	c.3815-1G>A		P/LP	1
**MYH7**	c.49C>T	p.Arg17Cys	VUS	2
**MYH7**	c.115G>A	p.Val39Met	VUS	1
**MYH7**	c.134A>G	p.Glu45Gly	VUS	1
**MYH7**	c.208A>T	p.Thr70Ser	P/LP	1
**MYH7**	c.293A>T	p.Glu98Val	VUS	1
**MYH7**	c.328G>T	p.Gly110Cys	VUS	1
**MYH7**	c.343T>C	p.Tyr115His	VUS	1
**MYH7**	c.350A>T	p.Tyr117Phe	P/LP	1
**MYH7**	c.428G>A	p.Arg143Gln	P/LP	6
**MYH7**	c.428G>C	p.Arg143Pro	VUS	1
**MYH7**	c.431G>A	p.Gly144Asp	VUS	1
**MYH7**	c.505A>G	p.Arg169Gly	P/LP	2
**MYH7**	c.507A>T	p.Arg169Ser	VUS	1
**MYH7**	c.530C>T	p.Thr177Ile	P/LP	1
**MYH7**	c.560A>G	p.Asn187Ser	VUS	1
**MYH7**	c.596C>T	p.Ala199Val	P/LP	1
**MYH7**	c.610C>T	p.Arg204Cys	VUS	1
**MYH7**	c.611G>A	p.Arg204His	P/LP	6
**MYH7**	c.619A>C	p.Lys207Gln	VUS	1
**MYH7**	c.664C>A	p.Gln222Lys	VUS	2
**MYH7**	c.676G>A	p.Ala226Thr	P/LP	1
**MYH7**	c.677C>T	p.Ala226Val	VUS	3
**MYH7**	c.697G>T	p.Ala233Ser	P/LP	2
**MYH7**	c.715G>A	p.Asp239Asn	P/LP	2
**MYH7**	c.717C>G	p.Asp239Glu	VUS	1
**MYH7**	c.727C>T	p.Arg243Cys	VUS	1
**MYH7**	c.740t>g	p.Phe247Cys	VUS	1
**MYH7**	c.746G>A	p.Arg249Gln	P/LP	7
**MYH7**	c.755T>G	p.Phe252Cys	P/LP	1
**MYH7**	c.788T>C	p.Ile263Thr	P/LP	9
**MYH7**	c.794C>A	p.Thr265Asn	VUS	1

**MYH7**	c.809A>G	p.Lys270Arg	P/LP	3
**MYH7**	c.830T>C	p.Leu277Pro	VUS	1
**MYH7**	c.872C>T	p.Ser291Phe	P/LP	2
**MYH7**	c.909C>G	p.Ile303Met	P/LP	1
**MYH7**	c.920C>A	p.Pro307His	P/LP	1
**MYH7**	c.925G>A	p.Asp309Asn	VUS	1
**MYH7**	c.950A>G	p.Glu317Gly	P/LP	1
**MYH7**	c.953C>A	p.Thr318Asn	VUS	2
**MYH7**	c.958G>A	p.Val320Met	P/LP	9
**MYH7**	c.968T>A	p.Ile323Asn	P/LP	4
**MYH7**	c.968T>C	p.Ile323Thr	VUS	1
**MYH7**	c.976G>C	p.Ala326Pro	VUS	6
**MYH7**	c.1012G>A	p.Val338Met	P/LP	4
**MYH7**	c.1013T>C	p.Val338Ala	P/LP	1
**MYH7**	c.1063G>A	p.Ala355Thr	P/LP	3
**MYH7**	c.1193G>A	p.Gly398Glu	P/LP	3
**MYH7**	c.1204C>T	p.Pro402Ser	VUS	1
**MYH7**	c.1207C>T	p.Arg403Trp	P/LP	4
**MYH7**	c.1208G>A	p.Arg403Gln	P/LP	15
**MYH7**	c.1273G>A	p.Gly425Arg	VUS	1
**MYH7**	c.1291G>C	p.Val431Leu	VUS	1
**MYH7**	c.1304T>G	p.Met435Arg	P/LP	1
**MYH7**	c.1324C>T	p.Arg442Cys	P/LP	5
**MYH7**	c.1331A>G	p.Asn444Ser	P/LP	2
**MYH7**	c.1357C>T	p.Arg453Cys	P/LP	9
**MYH7**	c.1358G>A	p.Arg453His	P/LP	2
**MYH7**	c.1370T>C	p.Ile457Thr	P/LP	2
**MYH7**	c.1405G>A	p.Asp469Asn	VUS	1
**MYH7**	c.1433T>A	p.Ile478Asn	VUS	1
**MYH7**	c.1479G>C	p.Met493Ile	P/LP	1
**MYH7**	c.1491G>T	p.Glu497Asp	P/LP	3
**MYH7**	c.1532T>C	p.Ile511Thr	VUS	3
**MYH7**	c.1588A>G	p.Ile530Val	P/LP	1
**MYH7**	c.1615A>C	p.Met539Leu	P/LP	2
**MYH7**	c.1711G>C	p.Gly571Arg	P/LP	2
**MYH7**	c.1727A>G	p.His576Arg	P/LP	3
**MYH7**	c.1742A>G	p.His581Arg	P/LP	2
**MYH7**	c.1750G>A	p.Gly584Ser	P/LP	3
**MYH7**	c.1750G>C	p.Gly584Arg	P/LP	12
**MYH7**	c.1801C>T	p.Leu601Phe	P/LP	1
**MYH7**	c.1807G>A	p.Glu603Lys	P/LP	1

**MYH7**	c.1816G>A	p.Val606Met	P/LP	8
**MYH7**	c.1820G>A	p.Gly607Asp	P/LP	3
**MYH7**	c.1826A>G	p.Tyr609Cys	P/LP	1
**MYH7**	c.1871A>G	p.Tyr624Cys	VUS	2
**MYH7**	c.1903A>T	p.Lys635*	VUS	1
**MYH7**	c.1906G>A	p.Gly636Ser	VUS	1
**MYH7**	c.1939G>A	p.Val647Leu	P/LP	1
**MYH7**	c.1954A>G	p.Arg652Gly	P/LP	7
**MYH7**	c.1987C>T	p.Arg663Cys	P/LP	18
**MYH7**	c.1988G>A	p.Arg663His	P/LP	22
**MYH7**	c.2069T>C	p.Met690Thr	P/LP	1
**MYH7**	c.2080C>T	p.Arg694Cys	P/LP	8
**MYH7**	c.2099A>C	p.Glu700Ala	VUS	1
**MYH7**	c.2104A>G	p.Ile702Val	P/LP	1
**MYH7**	c.2123G>C	p.Gly708Ala	P/LP	1
**MYH7**	c.2129C>A	p.Pro710His	P/LP	1
**MYH7**	c.2146G>A	p.Gly716Arg	P/LP	9
**MYH7**	c.2155C>T	p.Arg719Gly	P/LP	1
**MYH7**	c.2155C>T	p.Arg719Trp	P/LP	5
**MYH7**	c.2156G>A	p.Arg719Gln	P/LP	11
**MYH7**	c.2156G>C	p.Arg719Pro	P/LP	2
**MYH7**	c.2167C>G	p.Arg723Gly	P/LP	5
**MYH7**	c.2167C>T	p.Arg723Cys	P/LP	13
**MYH7**	c.2178C>A	p.Asn726Lys	VUS	1
**MYH7**	c.2183C>T	p.Ala728Val	VUS	1
**MYH7**	c.2190A>G	p.Ile730Met	P/LP	1
**MYH7**	c.2191C>G	p.Pro731Ala	P/LP	1
**MYH7**	c.2191C>T	p.Pro731Ser	P/LP	5
**MYH7**	c.2198G>A	p.Gly733Glu	P/LP	2
**MYH7**	c.2201A>C	p.Gln734Pro	P/LP	1
**MYH7**	c.2206A>G	p.Ile736Val	VUS	1
**MYH7**	c.2207T>C	p.Ile736Thr	P/LP	6
**MYH7**	c.2213G>C	p.Ser738Thr	VUS	1
**MYH7**	c.2221G>A	p.Gly741Arg	P/LP	3
**MYH7**	c.2221G>C	p.Gly741Arg	P/LP	4
**MYH7**	c.2221G>T	p.Gly741Trp	P/LP	1
**MYH7**	c.2291T>A	p.Phe764Tyr	P/LP	1
**MYH7**	c.2302G>A	p.Gly768Arg	P/LP	14
**MYH7**	c.2306T>C	p.Leu769Pro	P/LP	1
**MYH7**	c.2334C>G	p.Asp778Glu	VUS	2
**MYH7**	c.2346C>A	p.Ser782Arg	P/LP	5

**MYH7**	c.2347C>T	p.Arg783Cys	P/LP	1
**MYH7**	c.2389G>A	p.Ala797Thr	P/LP	18
**MYH7**	c.2424-5T>C		VUS	1
**MYH7**	c.2432T>C	p.Leu811Pro	P/LP	1
**MYH7**	c.2451C>A	p.Asn817Lys	VUS	1
**MYH7**	c.2471T>C	p.Val824Ala	P/LP	1
**MYH7**	c.2481G>T	p.Trp827Cys	VUS	1
**MYH7**	c.2482C>T	p.Pro828Ser	VUS	1
**MYH7**	c.2494C>T	p.Leu832Phe	VUS	1
**MYH7**	c.2497T>C	p.Tyr833His	VUS	3
**MYH7**	c.2508C>G	p.Ile836Met	VUS	1
**MYH7**	c.2524A>G	p.Ser842Gly	P/LP	1
**MYH7**	c.2525G>A	p.Ser842Asn	VUS	2
**MYH7**	c.2536G>A	p.Glu846Lys	VUS	1
**MYH7**	c.2539_2541del	p.Lys847del	P/LP	5
**MYH7**	c.2539A>G	p.Lys847Glu	P/LP	1
**MYH7**	c.2542_2544del	p.Glu848del	P/LP	1
**MYH7**	c.2543A>G	p.Glu848Gly	P/LP	1
**MYH7**	c.2548G>A	p.Ala850Thr	VUS	1
**MYH7**	c.2555T>C	p.Met852Thr	VUS	2
**MYH7**	c.2572C>A	p.Arg858Ser	P/LP	1
**MYH7**	c.2572C>G	p.Arg858Gly	VUS	1
**MYH7**	c.2572C>T	p.Arg858Cys	P/LP	1
**MYH7**	c.2573G>C	p.Arg858Pro	P/LP	1
**MYH7**	c.2593A>G	p.Lys865Glu	VUS	2
**MYH7**	c.2594A>G	p.Lys865Arg	P/LP	3
**MYH7**	c.2605C>T	p.Arg869Cys	VUS	1
**MYH7**	c.2606G>A	p.Arg869His	P/LP	42
**MYH7**	c.2608C>T	p.Arg870Cys	P/LP	1
**MYH7**	c.2609G>A	p.Arg870His	P/LP	3
**MYH7**	c.2623_2625del	p.Glu875del	P/LP	1
**MYH7**	c.2631G>T	p.Met877Ile	P/LP	1
**MYH7**	c.2644C>G	p.Gln882Glu	P/LP	3
**MYH7**	c.2655T>A	p.Asn885Lys	P/LP	1
**MYH7**	c.2681A>G	p.Glu894Gly	P/LP	3
**MYH7**	c.2699A>T	p.Asp900Val	VUS	1
**MYH7**	c.2707G>C	p.Glu903Gln	VUS	1
**MYH7**	c.2717A>G	p.Asp906Gly	P/LP	4
**MYH7**	c.2722C>G	p.Leu908Val	P/LP	8
**MYH7**	c.2723T>A	p.Leu908Gln	P/LP	1
**MYH7**	c.2723T>C	p.Leu908Pro	VUS	1

**MYH7**	c.2727C>G	p.Ile909Met	P/LP	2
**MYH7**	c.2739T>G	p.Ile913Met	VUS	1
**MYH7**	c.2770G>A	p.Glu924Lys	P/LP	2
**MYH7**	c.2779G>A	p.Glu927Lys	VUS	5
**MYH7**	c.2783A>T	p.Asp928Val	P/LP	2
**MYH7**	c.2788G>C	p.Glu930Gln	P/LP	2
**MYH7**	c.2846A>T	p.Glu949Val	VUS	1
**MYH7**	c.2858A>T	p.Asp953Val	VUS	1
**MYH7**	c.2893G>A	p.Glu965Lys	P/LP	2
**MYH7**	c.2974C>A	p.Leu992Met	P/LP	1
**MYH7**	c.3100-2A>C		P/LP	1
**MYH7**	c.3133C>T	p.Arg1045Cys	P/LP	4
**MYH7**	c.3134G>A	p.Arg1045His	P/LP	1
**MYH7**	c.3158G>A	p.Arg1053Gln	VUS	3
**MYH7**	c.3169G>A	p.Gly1057Ser	VUS	1
**MYH7**	c.3170G>A	p.Gly1057Asp	P/LP	1
**MYH7**	c.3245+3A>T		VUS	1
**MYH7**	c.3274G>A	p.Ala1092Thr	VUS	1
**MYH7**	c.3346G>A	p.Glu1116Lys	P/LP	1
**MYH7**	c.3358G>A	p.Glu1120Lys	P/LP	1
**MYH7**	c.3382G>A	p.Ala1128Thr	VUS	2
**MYH7**	c.3407G>A	p.Arg1136His	VUS	1
**MYH7**	c.3551A>T	p.Gln1184Leu	VUS	1
**MYH7**	c.3597C>G	p.Ser1199Arg	VUS	1
**MYH7**	c.3610G>C	p.Gly1204Arg	VUS	1
**MYH7**	c.3637G>A	p.Val1213Met	VUS	5
**MYH7**	c.3645G>C	p.Gln1215His	P/LP	1
**MYH7**	c.3701A>C	p.Asn1234Thr	VUS	1
**MYH7**	c.3830G>A	p.Arg1277Gln	VUS	1
**MYH7**	c.3900G>C	p.Gln1300His	VUS	3
**MYH7**	c.4031G>A	p.Arg1344Gln	P/LP	2
**MYH7**	c.4066G>A	p.Glu1356Lys	P/LP	4
**MYH7**	c.4078G>A	p.Val1360Ile	VUS	2
**MYH7**	c.4123T>C	p.Tyr1375His	P/LP	1
**MYH7**	c.4124A>G	p.Tyr1375Cys	VUS	1
**MYH7**	c.4130C>T	p.Thr1377Met	P/LP	10
**MYH7**	c.4159G>A	p.Glu1387Lys	VUS	1
**MYH7**	c.4208C>A	p.Ala1403Asp	VUS	1
**MYH7**	c.4210G>A	p.Val1404Met	VUS	1
**MYH7**	c.4258C>T	p.Arg1420Trp	VUS	2
**MYH7**	c.4259G>A	p.Arg1420Gln	VUS	1

**MYH7**	c.4283T>C	p.Leu1428Ser	VUS	1
**MYH7**	c.4327G>A	p.Asp1443Asn	VUS	1
**MYH7**	c.4363G>A	p.Glu1455Lys	VUS	1
**MYH7**	c.4402G>A	p.Glu1468Lys	VUS	1
**MYH7**	c.4402G>C	p.Glu1468Gln	VUS	1
**MYH7**	c.4519+1G>C		VUS	1
**MYH7**	c.4571A>G	p.His1524Arg	P/LP	1
**MYH7**	c.4644+24G>T		VUS	1
**MYH7**	c.4679G>A	p.Arg1560Gln	VUS	1
**MYH7**	c.4816C>T	p.Arg1606Cys	VUS	2
**MYH7**	c.4817G>A	p.Arg1606His	VUS	3
**MYH7**	c.4864C>T	p.Leu1622Phe	VUS	1
**MYH7**	c.4908C>T	p.Ala1636Ala	VUS	1
**MYH7**	c.4915G>A	p.Ala1639Thr	VUS	1
**MYH7**	c.4954-14C>G		VUS	1
**MYH7**	c.4954G>T	p.Asp1652Tyr	VUS	2
**MYH7**	c.4992C>A	p.Asn1664Lys	P/LP	1
**MYH7**	c.5119A>T	p.Ile1707Phe	VUS	1
**MYH7**	c.5134C>T	p.Arg1712Trp	P/LP	1
**MYH7**	c.5135G>A	p.Arg1712Gln	P/LP	10
**MYH7**	c.5156A>G	p.Gln1719Arg	P/LP	2
**MYH7**	c.5272G>T	p.Ala1758Ser	VUS	1
**MYH7**	c.5302G>A	p.Glu1768Lys	VUS	2
**MYH7**	c.5305C>A	p.Leu1769Met	P/LP	1
**MYH7**	c.5329G>A	p.Ala1777Thr	VUS	2
**MYH7**	c.5341C>T	p.Arg1781Cys	VUS	1
**MYH7**	c.5342G>A	p.Arg1781His	VUS	3
**MYH7**	c.5344A>G	p.Met1782Val	P/LP	1
**MYH7**	c.5422G>A	p.Gly1808Ser	P/LP	1
**MYH7**	c.5500G>A	p.Ala1834Thr	VUS	1
**MYH7**	c.5527A>G	p.Ser1843Gly	VUS	1
**MYH7**	c.5606A>G	p.Asp1869Gly	P/LP	1
**MYH7**	c.5647G>A	p.Glu1883Lys	VUS	1
**MYH7**	c.5696T>C	p.Val1899Ala	P/LP	1
**MYL2**	c.1A>G	p.Met1?	VUS	1
**MYL2**	c.45_46delinsT	p.Asn16Thrfs*34	P/LP	1
**MYL2**	c.49G>A	p.Val17Met	VUS	1
**MYL2**	c.58A>C	p.Met20Leu	P/LP	4
**MYL2**	c.64G>A	p.Glu22Lys	P/LP	6
**MYL2**	c.119G>A	p.Arg40Lys	VUS	1

**MYL2**	c.125G>A	p.Gly42Asp	P/LP	2
**MYL2**	c.173G>A	p.Arg58Gln	P/LP	8
**MYL2**	c.184A>T	p.Lys62*	P/LP	1
**MYL2**	c.193G>A	p.Glu65Lys	P/LP	1
**MYL2**	c.239C>A	p.Thr80Asn	P/LP	2
**MYL2**	c.313G>A	p.Val105Met	VUS	1
**MYL2**	c.401A>C	p.Glu134Ala	VUS	5
**MYL2**	c.459G>C	p.Lys153Asn	VUS	1
**MYL2**	c.482A>G	p.His161Arg	P/LP	1
**MYL2**	c.484G>A	p.Gly162Arg	P/LP	1
**MYL3**	c.170C>A	p.Ala57Asp	VUS	3
**MYL3**	c.170C>G	p.Ala57Gly	P/LP	1
**MYL3**	c.184G>A	p.Asp62Asn	VUS	1
**MYL3**	c.427G>A	p.Glu143Lys	VUS	2
**MYL3**	c.445A>G	p.Met149Val	P/LP	1
**MYL3**	c.452C>T	p.Ala151Val	P/LP	3
**MYL3**	c.466G>A	p.Val156Met	VUS	1
**MYL3**	c.466G>T	p.Val156Leu	VUS	2
**MYL3**	c.517A>G	p.Met173Val	VUS	1
**MYL3**	c.530A>G	p.Glu177Gly	VUS	1
**TNNI3**	c.115dup	p.Ser39Phefs*2	P/LP	1
**TNNI3**	c.257_258del	p.Ala86Glyfs*23	P/LP	2
**TNNI3**	c.302A>G	p.His101Arg	VUS	1
**TNNI3**	c.356C>A	p.Thr119Asn	P/LP	1
**TNNI3**	c.370G>C	p.Glu124Gln	VUS	1
**TNNI3**	c.407G>A	p.Arg136Gln	P/LP	1
**TNNI3**	c.422G>A	p.Arg141Gln	P/LP	1
**TNNI3**	c.428C>A	p.Thr143Asn	VUS	2
**TNNI3**	c.433C>G	p.Arg145Gly	P/LP	1
**TNNI3**	c.433C>T	p.Arg145Trp	P/LP	5
**TNNI3**	c.434G>A	p.Arg145Gln	P/LP	1
**TNNI3**	c.470C>T	p.Ala157Val	P/LP	1
**TNNI3**	c.481G>C	p.Ala161Pro	VUS	1
**TNNI3**	c.484C>T	p.Arg162Trp	P/LP	1
**TNNI3**	c.485G>A	p.Arg162Gln	P/LP	6
**TNNI3**	c.491A>C	p.Lys164Thr	VUS	1
**TNNI3**	c.497C>T	p.Ser166Phe	P/LP	3
**TNNI3**	c.526G>A	p.Val176Met	P/LP	1
**TNNI3**	c.549G>T	p.Lys183Asn	P/LP	1

**TNNI3**	c.557G>A	p.Arg186Gln	P/LP	10
**TNNI3**	c.568G>T	p.Asp190Tyr	P/LP	1
**TNNI3**	c.575G>T	p.Arg192Leu	P/LP	1
**TNNI3**	c.581A>G	p.Asn194Ser	P/LP	1
**TNNI3**	c.586G>A	p.Asp196Asn	VUS	1
**TNNI3**	c.592C>G	p.Leu198Val	P/LP	1
**TNNI3**	c.592C>G	p.Leu198Val	P/LP	6
**TNNI3**	c.596G>A	p.Ser199Asn	VUS	1
**TNNT2**	c.68-3del		VUS	1
**TNNT2**	c.68-5C>T		VUS	1
**TNNT2**	c.196A>C	p.Lys66Gln	P/LP	1
**TNNT2**	c.236T>A	p.Ile79Asn	P/LP	6
**TNNT2**	c.274C>T	p.Arg92Trp	P/LP	6
**TNNT2**	c.275G>A	p.Arg92Gln	P/LP	5
**TNNT2**	c.280C>T	p.Arg94Cys	P/LP	1
**TNNT2**	c.281G>A	p.Arg94His	P/LP	1
**TNNT2**	c.291G>T	p.Lys97Asn	VUS	1
**TNNT2**	c.299A>G	p.Asn100Ser	VUS	1
**TNNT2**	c.305G>A	p.Arg102Gln	P/LP	1
**TNNT2**	c.311C>T	p.Ala104Val	VUS	1
**TNNT2**	c.330T>G	p.Phe110Leu	P/LP	5
**TNNT2**	c.388C>T	p.Arg130Cys	P/LP	4
**TNNT2**	c.420C>T	p.Arg140=	VUS	1
**TNNT2**	c.421del	p.Arg141fs	P/LP	1
**TNNT2**	c.487_489del	p.Glu163del	P/LP	4
**TNNT2**	c.520A>T	p.Asn174Tyr	P/LP	1
**TNNT2**	c.534G>C	p.Leu178Phe	VUS	2
**TNNT2**	c.571-1G>A		VUS	1
**TNNT2**	c.583G>A	p.Glu195Lys	P/LP	1
**TNNT2**	c.653T>C	p.Ile218Thr	P/LP	1
**TNNT2**	c.744C>T	p.Phe248=	VUS	1
**TNNT2**	c.773A>T	p.Lys258Ile	VUS	3
**TNNT2**	c.785A>G	p.Asn262Ser	VUS	2
**TNNT2**	c.803A>T	p.Asn268Ile	P/LP	1
**TNNT2**	c.805A>G	p.Asn269Asp	VUS	1
**TNNT2**	c.807C>A	p.Asn269Lys	P/LP	1
**TNNT2**	c.843-23A>G		VUS	1
**TNNT2**	c.853C>T	p.Arg285Cys	P/LP	7
**TNNT2**	c.856C>T	p.Arg286Cys	P/LP	7
**TNNT2**	c.860G>A	p.Trp287*	P/LP	6

**TNNT2**	c.877C>T	p.Arg293Cys	P/LP	1
**TNNT2**	c.881G>A	p.Trp294*	P/LP	2
**TPM1**	c.58G>A	p.Asp20Asn	VUS	1
**TPM1**	c.62G>T	p.Arg21Leu	P/LP	1
**TPM1**	c.64G>A	p.Ala22Thr	P/LP	2
**TPM1**	c.69G>C	p.Glu23Asp	VUS	1
**TPM1**	c.73G>A	p.Ala25Thr	P/LP	1
**TPM1**	c.184G>T	p.Glu62Gln	P/LP	2
**TPM1**	c.198G>C	p.Lys66Asn	VUS	1
**TPM1**	c.368G>A	p.Ser123Asn	VUS	2
**TPM1**	c.428T>G	p.Ile143Ser	P/LP	1
**TPM1**	c.457C>G	p.His153Asp	P/LP	2
**TPM1**	c.523G>A	p.Asp175Asn	P/LP	2
**TPM1**	c.574G>A	p.Glu192Lys	P/LP	4
**TPM1**	c.644C>T	p.Ser215Leu	VUS	2
**TPM1**	c.655G>A	p.Asp219Asn	P/LP	1
**TPM1**	c.761A>G	p.Asp254Gly	VUS	1
**TPM1**	c.761A>G	p.Asp254Gly	VUS	1
**TPM1**	c.762T>G	p.Asp254Glu	VUS	1
**TPM1**	c.773-3T>C		VUS	1
**TPM1**	c.775A>G	p.Lys258Glu	VUS	1
**TPM1**	c.832C>T	p.Leu278Phe	VUS	1
**TPM1**	c.842T>C	p.Met281Thr	P/LP	1
**TPM1**	c.850A>G	p.Ile284Val	VUS	4

† Splice site mutation

Supplemental Table 2. Annualized risk of the overall composite outcome estimated based on time from birth and time from diagnosis in the Genotyped HCM Cohort.

**Time from birth**				
**Group**	**# of patients**	**# of events**	**IR per 1000 p-y**	**Annualized risk (%)**
All patients	2637	1140	8.64	0.86
Age at diagnosis <40 years	1100	410	10.3	1.03
Age at diagnosis 40-60 years	1041	454	7.91	0.79
Age at diagnosis >60 years	496	276	7.93	0.79

**Time from diagnosis (all events)**				
**Group**	**# of patients**	**# of events**	**IR per 1000 p-y**	**Annualized risk (%)**
All patients	2462	985	50.2	5.02
Age at diagnosis <40 years	1065	386	31.5	3.15
Age at diagnosis 40-60 years	973	393	66.5	6.65
Age at diagnosis >60 years	424	206	141.9	14.19

**Time from diagnosis (incident events only)**				
**Group**	**# of patients**	**# of events**	**IR per 1000 p-y**	**Annualized risk (%)**
All patients	2371	894	45.6	4.56
Age at diagnosis <40 years	1040	361	29.5	2.95
Age at diagnosis 40-60 years	931	351	59.4	5.94
Age at diagnosis >60 years	400	182	125.4	12.54

Supplemental Table 3. Multiple sarcomere mutations predict higher risk of adverse outcomes

Overall Composite

	Hazard Ratio	Lower 95%	Upper 95%	P-value
SARC +	1.53	1.30	1.80	<0.001
SARC 2+	2.54	1.63	3.96	<0.01
SARC VUS	1.41	1.08	1.83	<0.05
Family Proband	1.69	1.31	2.17	<0.001
Female	0.94	0.81	1.095	0.41
Non-White	1.10	0.86	1.42	0.45
Diagnosis Age <40 years	8.33	6.94	8.93	<0.001

Number of Events	Number of Patients
1125	2579

Ventricular Arrhythmia Composite

	Hazard Ratio	Lower 95%	Upper 95%	P-value
SARC +	1.76	1.25	2.49	<0.01
SARC 2+	4.15	1.96	8.76	<0.001
SARC VUS	1.28	0.73	2.26	0.40
Family Proband	5.99	2.42	14.79	<0.001
Female	0.72	0.53	0.99	<0.05
Non-White	1.67	1.04	2.66	<0.05
Diagnosis Age <40 years	6.99	4.90	10.00	<0.001

Number of Events	Number of Patients
186	2633

Heart Failure Composite

	Hazard Ratio	Lower 95%	Upper 95%	P-value
SARC +	1.39	1.13	1.71	<0.01
SARC 2+	2.17	1.23	3.72	<0.01
SARC VUS	1.16	0.82	1.64	0.39
Family Proband	1.62	1.17	1.15	<0.01
Female	1.41	1.18	1.69	<0.001
Non-White	1.05	0.76	1.45	0.75
Diagnosis Age <40 years	6.10	4.87	7.63	<0.001

Number of Events	Number of Patients
719	2639

Atrial Fibrillation

	Hazard Ratio	Lower 95%	Upper 95%	P-value
SARC +	1.73	1.40	2.15	<0.001
SARC 2+	1.90	1.01	3.58	<0.05
SARC VUS	1.70	1.21	2.39	<0.01
Family Proband	1.62	1.17	2.25	<0.01
Female	0.78	0.64	0.94	<0.05
Non-White	1.02	0.73	1.44	0.90
Diagnosis Age <40 years	6.71	5.29	8.47	<0.001

Number of Events	Number of Patients
593	2537

Supplemental Table 4. Baseline characteristics for patients censored before and after 2011

	Total	Overall Composite: censored before 2011 (likely lost to follow-up) [A]	Overall Composite: censored 2011 or after [B]	Overall Composite: event occurred [C]	P-value ([A] vs. [B])
	N = 4,209	N = 747	N = 1,667	N = 1,795	
**DEMOGRAPHICS**					
Age at Diagnosis (years)					0.37
Mean ± SD	44.24 ± 18.49	41.54 ± 17.63	42.26 ± 18.34	47.20 ± 18.57	
Median	45.73	42.71	43.98	49.16	
IQR	(30.89, 58.08)	(28.44, 54.25)	(28.00, 55.24)	(34.98, 61.32)	
Range	(0.00, 94.33)	(0.00, 89.20)	(0.00, 89.45)	(0.00, 94.33)	
Missing / N (%)	0 / 4,209 (0.00)	0 / 747 (0.00)	0 / 1,667 (0.00)	0 / 1,795 (0.00)	
Age at First Encounter (years)					0.63
Mean ± SD	47.36 ± 17.71	43.94 ± 17.05	44.31 ± 17.93	51.60 ± 16.88	
Median	48.92	45.39	45.96	53.19	
IQR	(35.00, 60.71)	(32.02, 56.83)	(30.97, 57.61)	(41.00, 64.41)	
Range	(0.00, 94.39)	(0.00, 89.20)	(0.04, 89.45)	(1.44, 94.39)	
Missing / N (%)	0 / 4,209 (0.00)	0 / 747 (0.00)	0 / 1,667 (0.00)	0 / 1,795 (0.00)	
Follow-up Time (years)					< 0.001
Mean ± SD	5.46 ± 7.06	3.45 ± 5.80	4.87 ± 6.10	6.85 ± 8.01	
Median	2.87	0.83	2.81	4.12	
IQR	(0.31, 7.99)	(0.00, 4.30)	(0.29, 7.28)	(0.83, 9.86)	

Range	(0.00, 49.44)	(0.00, 34.84)	(0.00, 38.54)	(0.00, 49.44)	
Missing / N (%)	0 / 4,209 (0.00)	0 / 747 (0.00)	0 / 1,667 (0.00)	0 / 1,795 (0.00)	
Gender					< 0.05
Female	1,566 (37.21%)	216 (28.92%)	571 (34.25%)	779 (43.40%)	
Male	2,643 (62.79%)	531 (71.08%)	1,096 (65.75%)	1,016 (56.60%)	
Missing / N (%)	0 / 4,209 (0.00%)	0 / 747 (0.00%)	0 / 1,667 (0.00%)	0 / 1,795 (0.00%)	
Race					< 0.05
Asian	93 (2.28%)	11 (1.50%)	47 (2.96%)	35 (1.99%)	
Black	127 (3.11%)	10 (1.36%)	56 (3.53%)	61 (3.47%)	
More than One	13 (0.32%)	2 (0.27%)	5 (0.32%)	6 (0.34%)	
Other or Not Reported	199 (4.88%)	44 (5.99%)	86 (5.42%)	69 (3.92%)	
White	3,628 (88.90%)	662 (90.07%)	1,386 (87.33%)	1,580 (89.82%)	
Missing / N (%)	128 / 4,209 (3.04%)	12 / 747 (1.61%)	80 / 1,667 (4.80%)	36 / 1,795 (2.01%)	
Pediatric Status	274 (6.51%)	55 (7.36%)	155 (9.30%)	64 (3.57%)	0.14
Missing / N (%)	0 / 4,209 (0.00%)	0 / 747 (0.00%)	0 / 1,667 (0.00%)	0 / 1,795 (0.00%)	
SCD Family History	864 (38.30%)	133 (50.76%)	342 (35.40%)	389 (37.84%)	< 0.001
Missing / N (%)	1,953 / 4,209 (46.40%)	485 / 747 (64.93%)	701 / 1,667 (42.05%)	767 / 1,795 (42.73%)	
HCM Family History	1,526 (36.26%)	270 (36.14%)	627 (37.61%)	629 (35.04%)	0.52
Missing / N (%)	0 / 4,209 (34.88%)	0 / 747 (0.00%)	0 / 1,667 (0.00%)	0 / 1,795 (0.00%)	
Age of Diagnosis Group					0.3
<40	1,640 (38.96%)	339 (45.38%)	703 (42.17%)	598 (33.31%)	
40-60	1,660 (39.44%)	288 (38.55%)	668 (40.07%)	704 (39.22%)	
>60	909 (21.60%)	120 (16.06%)	296 (17.76%)	493 (27.47%)	
Missing / N (%)	0 / 4,209 (0.00%)	0 / 747 (0.00%)	0 / 1,667 (0.00%)	0 / 1,795 (0.00%)	

**GENTETIC CHARACTERISTICS**					
Sarcomere Group					0.1
SARC(-)	1,181 (44.79%)	206 (48.93%)	462 (42.94%)	513 (45.00%)	
SARC(+)	1,225 (46.45%)	183 (43.47%)	516 (47.96%)	526 (46.14%)	
SARC(U)	231 (8.76%)	32 (7.60%)	98 (9.11%)	101 (8.86%)	
Missing / N (%)	1,572 / 4,209 (37.35%)	326 / 747 (43.64%)	591 / 1,667 (35.45%)	655 / 1,795 (36.49%)	
**CLINICAL CHARACTERISTICS**					
Family Proband	3,691 (87.69%)	643 (86.08%)	1,415 (84.88%)	1,633 (90.97%)	0.48
Missing / N (%)	0 / 4,209 (0.00%)	0 / 747 (0.00%)	0 / 1,667 (0.00%)	0 / 1,795 (0.00%)	
Age at First Clinic Visit (years)					0.35
Mean ± SD	47.87 ± 17.73	44.15 ± 17.01	44.88 ± 17.97	52.19 ± 16.85	
Median	49.3	45.64	46.59	53.73	
IQR	(35.55, 61.12)	(32.50, 56.70)	(31.89, 58.10)	(41.78, 64.95)	
Range	(0.00, 94.39)	(0.00, 89.20)	(0.04, 89.45)	(1.44, 94.39)	
Missing / N (%)	37 / 4,209 (0.88)	20 / 747 (2.68)	1 / 1,667 (0.06)	16 / 1,795 (0.89)	
**ECHOCARDIOGRAPHIC CHARACTERISTICS**					
Age at First Echo (years)					0.68
Mean ± SD	48.58 ± 17.44	44.96 ± 17.02	45.28 ± 17.68	53.17 ± 16.32	
Median	50.15	46.33	46.88	54.77	
IQR	(36.54, 61.51)	(33.33, 57.94)	(32.96, 58.53)	(43.24, 65.23)	
Range	(0.00, 94.39)	(0.00, 89.20)	(0.04, 89.45)	(1.98, 94.39)	
Missing / N (%)	0 / 4,209 (0.00)	0 / 747 (0.00)	0 / 1,667 (0.00)	0 / 1,795 (0.00)	
LA Diameter (mm)					< 0.001
Mean ± SD	45.22 ± 9.86	44.44 ± 10.43	41.94 ± 7.89	48.79 ± 10.18	
Median	44	43	42	48	

IQR	(39.00, 50.00)	(37.00, 50.00)	(37.00, 47.00)	(42.00, 54.00)	
Range	(5.80, 100.00)	(5.80, 80.00)	(18.00, 76.00)	(15.00, 100.00)	
Missing / N (%)	930 / 4,209 (22.10)	180 / 747 (24.10)	318 / 1,667 (19.08)	432 / 1,795 (24.07)	
LVEF (%)					0.35
Mean ± SD	64.93 ± 9.60	65.59 ± 8.28	65.96 ± 7.86	63.61 ± 11.38	
Median	65	65	65	65	
IQR	(60.00, 71.00)	(60.00, 71.00)	(60.00, 71.00)	(60.00, 71.00)	
Range	(15.00, 92.00)	(22.00, 90.00)	(32.00, 91.00)	(15.00, 92.00)	
Missing / N (%)	740 / 4,209 (17.58)	180 / 747 (24.10)	201 / 1,667 (12.06)	359 / 1,795 (20.00)	
Maximal LVWT (mm)					< 0.001
Mean ± SD	18.86 ± 5.64	19.11 ± 5.60	17.68 ± 5.43	19.85 ± 5.66	
Median	18	18	17	19	
IQR	(15.00, 22.00)	(15.00, 22.00)	(14.00, 21.00)	(16.00, 23.00)	
Range	(3.60, 45.00)	(7.00, 43.00)	(3.60, 45.00)	(5.60, 44.00)	
Missing / N (%)	84 / 4,209 (2.00)	13 / 747 (1.74)	26 / 1,667 (1.56)	45 / 1,795 (2.51)	
Doppler Obstruction	1,293 (30.72%)	171 (22.89%)	448 (26.87%)	674 (37.55%)	< 0.05
Missing / N (%)	0 / 4,209 (0.00%)	0 / 747 (0.00%)	0 / 1,667 (0.00%)	0 / 1,795 (0.00%)	

Supplemental Table 5. **Cox proportional hazards models analyzing appropriate implantable cardiovertor-defibrillator (ICD) therapies, including and excluding events that consisted only of antitachycardia pacing (ATP).**

Fitted Cox Proportional Hazard Model for Ventricular Arrhythmia Composite (All ICD therapies)			
	**Hazard Ratio**	**95% Cl**	**p-value**
**Sarcomere Group vs SARC -**			
**SARC +**	2.35	(1.73, 3.20)	<0.001
**SARC VUS**	1.65	(0.97, 2.81)	0.07

Fitted Cox Proportional Hazard Model for Ventricular Arrhythmia Composite (Excluding therapies consisting only of ATP)			
**Sarcomere Group vs SARC -**			
**SARC +**	2.36	(1.71, 3.25)	<0.001
**SARC VUS**	1.7	(0.98, 2.96)	0.06

REFERENCES FOR SUPPLEMENTAL MATERIAL

1. Lek M, Karczewski KJ, Minikel EV, Samocha KE, Banks E, Fennell T, O'Donnell-Luria AH, Ware JS, Hill AJ, Cummings BB, Tukiainen T, Birnbaum DP, Kosmicki JA, Duncan LE, Estrada K, Zhao F, Zou J, Pierce-Hoffman E, Berghout J, Cooper DN, Deflaux N, DePristo M, Do R, Flannick J, Fromer M, Gauthier L, Goldstein J, Gupta N, Howrigan D, Kiezun A, Kurki MI, Moonshine AL, Natarajan P, Orozco L, Peloso GM, Poplin R, Rivas MA, Ruano-Rubio V, Rose SA, Ruderfer DM, Shakir K, Stenson PD, Stevens C, Thomas BP, Tiao G, Tusie-Luna MT, Weisburd B, Won HH, Yu D, Altshuler DM, Ardissino D, Boehnke M, Danesh J, Donnelly S, Elosua R, Florez JC, Gabriel SB, Getz G, Glatt SJ, Hultman CM, Kathiresan S, Laakso M, McCarroll S, McCarthy MI, McGovern D, McPherson R, Neale BM, Palotie A, Purcell SM, Saleheen D, Scharf JM, Sklar P, Sullivan PF, Tuomilehto J, Tsuang MT, Watkins HC, Wilson JG, Daly MJ, MacArthur DG. Analysis of protein-coding genetic variation in 60,706 humans. *Nature*. 2016;536:285-291.
2. Whiffin N, Walsh R, Govind R, Edwards M, Ahmad M, Zhang X, Tayal U, Buchan R, Midwinter W, Wilk AE, Najgebauer H, Francis C, Wilkinson S, Monk T, Brett L, O'Regan DP, Prasad SK, Morris-Rosendahl DJ, Barton PJR, Edwards E, Ware JS, Cook SA. Cardioclassifier: Disease- and gene-specific computational decision support for clinical genome interpretation. *Genet Med*. 2018 DOI 10.1038/gim.2017.258.
3. Walsh R, Thomson KL, Ware JS, Funke BH, Woodley J, McGuire KJ, Mazzarotto F, Blair E, Seller A, Taylor JC, Minikel EV, Exome Aggregation C, MacArthur DG, Farrall M, Cook SA, Watkins H. Reassessment of mendelian gene pathogenicity using 7,855 cardiomyopathy cases and 60,706 reference samples. *Genet Med*. 2017;19:192-203.
